# The Non-amyloidal Component Region of α-Synuclein Is Important for α-Synuclein Transport Within Axons

**DOI:** 10.3389/fncel.2019.00540

**Published:** 2020-01-10

**Authors:** Eric N. Anderson, Delnessaw Hirpa, Kan Hong Zheng, Rupkatha Banerjee, Shermali Gunawardena

**Affiliations:** Department of Biological Sciences, The State University of New York at Buffalo, Buffalo, NY, United States

**Keywords:** α-synuclein (a-synuclein), axonal transport disruption, Parkinson’s disease, synaptic abnormality, *in vivo* imaging

## Abstract

Proper transport of the Parkinson’s disease (PD) protein, α-synuclein (α-syn), is thought to be crucial for its localization and function at the synapse. Previous work has shown that defects in long distance transport within narrow caliber axons occur early in PD, but how such defects contribute to PD is unknown. Here we test the hypothesis that the NAC region is involved in facilitating proper transport of α-syn within axons *via* its association with membranes. Excess α-syn or fPD mutant α-syn^A53T^ accumulates within larval axons perturbing the transport of synaptic proteins. These α-syn expressing larvae also show synaptic morphological and larval locomotion defects, which correlate with the extent of α-syn-mediated axonal accumulations. Strikingly, deletion of the NAC region (α-syn^Δ71–82^) prevented α-syn accumulations and axonal blockages, and reduced its synaptic localization due to decreased axonal entry and axonal transport of α-syn, due to less α-syn bound to membranes. Intriguingly, co-expression α-syn^Δ71–82^ with full-length α-syn rescued α-syn accumulations and synaptic morphological defects, and decreased the ratio of the insoluble higher molecular weight (HMW)/soluble low molecular weight (LMW) α-syn, indicating that this region is perhaps important for the dimerization of α-syn on membranes. Together, our observations suggest that under physiological conditions, α-syn associates with membranes *via* the NAC region, and that too much α-syn perturbs axonal transport *via* aggregate formation, instigating synaptic and behavioral defects seen in PD.

## Graphical Abstract

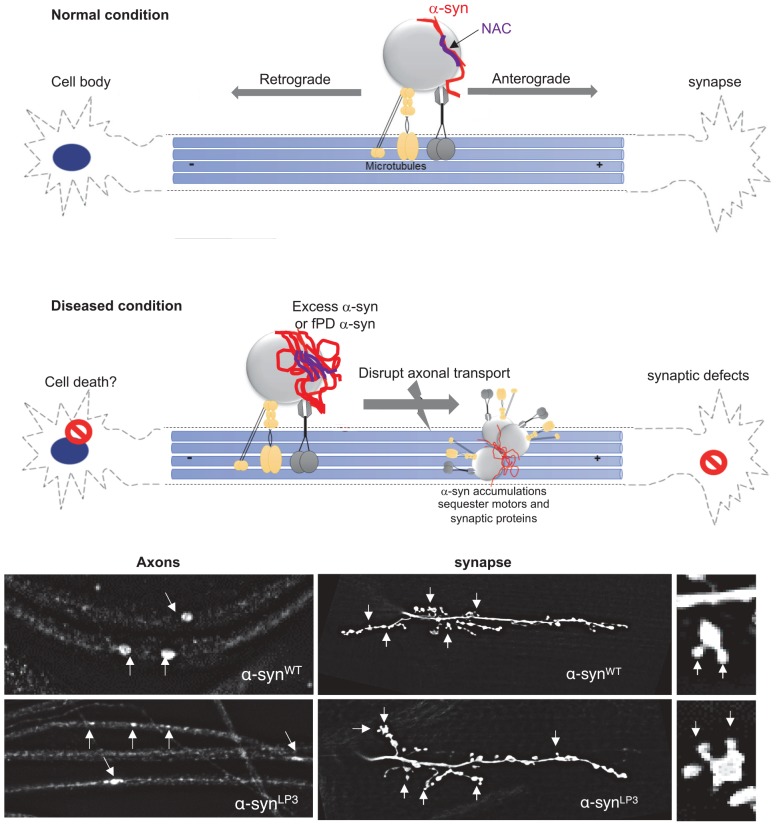

## Introduction

Parkinson’s disease (PD) is a common neurodegenerative disease characterized by loss of dopaminergic (DA) neurons in the substanita nigra pars compacta (SNpc) (Dawson and Dawson, 2003; Hardy et al., 2009). The most common histopathological characteristic of PD is the formation of α-synuclein (α-syn)-containing inclusions called Lewy bodies (LBs). α-Syn is a small acidic protein composed of 140 amino acid residues (Ueda et al., 1993). It is a soluble, natively unfolded protein, which likely becomes structured upon binding to phospholipid vesicles (Davidson et al., 1998; Eliezer et al., 2001; Li et al., 2001). The α-syn protein contains three distinct domains; a highly conserved amino terminal amphipathic α-helical domain, which is thought to associate with membranes (Ueda et al., 1993), a central hydrophobic region known as the non-amyloidal component (NAC) which is proposed to be essential for α-syn aggregation, and an acidic carboxyl-terminal domain, which is suggested to have chaperone-like activity (Ueda et al., 1993; Giasson et al., 2001). Three missense mutations (A53T, E46K, and A30P), located in the amphipathic α-helical domain, as well as duplication and triplication of the α-syn gene are implicated in familial PD (fPD) (Polymeropoulos et al., 1997), with increased levels of α-syn causing α-syn aggregation leading to neuronal dysfunction (Masliah et al., 2000; Giasson et al., 2002; Zach et al., 2007; Jiang et al., 2010; Spinelli et al., 2014).

In the central nervous system (CNS), α-syn associates with vesicles and lipids (Davidson et al., 1998; Jensen et al., 1998; Rhoades et al., 2006) and is enriched in presynaptic terminals (Maroteaux et al., 1988). Many roles for α-syn, such as neurotransmitter release (Chandra et al., 2005; Burré et al., 2010), synaptic vesicle trafficking (Lee et al., 2011), and axonal transport (Jensen et al., 1998; Roy et al., 2000) have been proposed. However, the physiological function of α-syn still remains elusive and is compounded by the fact that α-syn knockout mice do not show aberrant phenotypes, is fertile, viable, and has normal brain morphology (Abeliovich et al., 2000). While α-syn is known to be predominantly transported within axons in the slow axonal component-b (SCb, rate of ∼2–8 mm/day) together with SCb proteins, synapsin-1, and GAPDH (Roy et al., 2007), it can also be moved in the fast axonal component with synaptophysin (FC, rate of 50–400 mm/day) (Jensen et al., 1998, 1999; Roy et al., 2007). Interestingly, while associations between α-syn and molecular motors kinesin-1 and dynein have been shown (Utton et al., 2005), how defects in the axonal transport of α-syn contribute to PD pathology is unclear.

In sporadic PD patient brains, axonal swellings contained phosphorylated α-syn (Coleman, 2005; Chung et al., 2009; Chu et al., 2012; Lundblad et al., 2012) with decreased levels of motor proteins (Chu et al., 2012). The rate of α-syn transport in the SC also appeared to decrease with age (Li et al., 2003), and fPD mutations A30P and A53T exhibited reduced transport in cultured neurons (Saha et al., 2004; Roy, 2009). While these observations suggest that the axonal transport pathway and α-syn biology are likely linked, the mechanistic details of how α-syn utilizes molecular motors for its motility are unclear. Since previous work has implicated the NAC region of α-syn in membrane association (Fusco et al., 2014), here we test the hypothesis that the NAC region is involved in the axonal motility of α-syn *in vivo.* We found that excess human α-syn or the fPD mutant α-syn^A53T^ cause α-syn accumulations that disrupt axonal transport. The axonal motility of and the membrane binding of α-syn is dependent on the NAC region, as deletion of the NAC region (α-syn^Δ71–82^) eliminated axonal accumulations and decreased α-syn binding to membranes. Further, expression of α-syn^Δ71–82^ with full-length α-syn decreased α-syn accumulations and synaptic morphological defects perhaps by decreasing the insoluble higher molecular weight (HMW) α-syn fraction. Collectively, our findings suggest that under physiological conditions, the NAC region is likely required for membrane binding and for the axonal motility of α-syn. Our observations also propose that α-syn-mediated axonal transport defects caused by excess or fPD mutant α-syn can instigate the synaptic abnormalities and behavioral phenotypes observed in PD.

## Results

### Excess α-Synuclein Causes Axonal Accumulates and Disrupts Axonal Transport in *Drosophila* Larval Segmental Nerves

**FIGURE 1:**
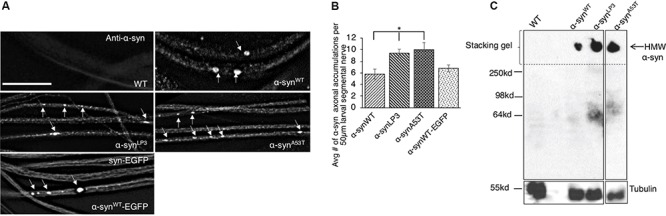
Excess human α-syn accumulates in larval axons *in vivo* and forms higher molecular weight α-syn species. **(A)** Expression of human wild-type α-syn (α-syn^WT^), a human α-syn line that expresses at higher levels (α-syn^LP3^), fPD α-syn mutant A53T (α-syn^A53T^), or EGFP tagged wild type α-syn (α-syn^WT^-EGFP) in larval segmental nerves show α-syn accumulations (arrows). Wild-type (WT) larvae do not stain for α-syn. **(B)** Quantitative analysis of the average number (#) of α-syn accumulations per 50 μm larval segmental nerve shows a significantly higher number in α-syn^LP3^ (*p* < 0.05) or α-syn^A53T^ (*p* < 0.05) larval axons compared to α-syn^WT^. Note that α-syn^WT^-EGFP and α-syn^WT^ contain similar amounts of α-syn accumulations. Quantitative analysis represents mean ± SEM. ^∗^*p* < 0.05, *N* = 7, bar = 25 μm. **(C)** Western blots from native non-denaturing gel electrophoresis reveal higher molecular weight (HMW) insoluble α-syn (arrow) in the stacking gel. Note that a α-syn smear is seen at approximately 64 kDa (low molecular weight), which is may represent soluble α-syn conformers. Tubulin is used as a loading control.

To test the prediction that excess α-syn causes α-syn accumulations within axons which disrupt the transport of essential synaptic proteins to synapses, we utilized a simple genetic model system where the physiological properties of α-syn can be investigated without the expression of endogenous α-syn. The *Drosophila* genome lacks an α-syn gene; however, *Drosophila* models overexpressing wild-type (WT) or PD-linked mutant forms of human α-syn show several features of PD including loss of dopaminergic neurons, LB-like filamentous intraneuronal inclusions, and locomotor dysfunction (Feany and Bender, 2000; Trinh et al., 2008), indicating that the cellular and molecular pathways influenced by excess α-syn are conserved in the fly. We expressed WT human α-syn (α-syn^WT^), a human α-syn line that expresses at higher levels (α-syn^LP3^, Trinh et al., 2008), and an fPD α-syn mutant (α-syn^A53T^) in *Drosophila* larval segmental nerves using the pan neuronal GAL4 driver, APPLGAL4 (Gunawardena and Goldstein, 2001; Gunawardena et al., 2003). We found α-syn-positive axonal accumulations within larval segmental nerves (Figure 1A). Quantification analysis indicates that the extent of axonal accumulations in larvae expressing α-syn^LP3^ or α-syn^A53T^ is significantly higher than what was seen in larvae expressing α-syn^WT^ (Figures 1A,B, *p* < 0.05). SDS-page and Western blot analysis revealed the extent of α-syn protein expression in larval brains, and confirmed that α-syn^LP3^ had an increased level of α-syn expression compared to α-syn^WT^ and α-syn^A53T^ (Supplementary Figures S1A,B, *p* < 0.01). Expression of the EGFP-tagged WT human α-syn, α-syn^WT^-EGFP (Poças et al., 2014) also showed axonal accumulations at levels comparable to α-syn^WT^. These α-syn^WT^-EGFP accumulations co-localized with an antibody against human α-syn (Supplementary Figure S1C) and a phospho-serine/threonine antibody (Supplementary Figure S2C). Therefore, overexpression of α-syn or fPD mutant α-syn causes α-syn-axonal accumulations which likely contain phosphorylated α-syn.

Previous studies showed that excess α-syn can accumulate into HMW insoluble aggregates termed LBs (Periquet et al., 2007). Therefore, we next evaluated the state of α-syn’s structure using native non-denaturing gel electrophoresis. Indeed, we found that flies expressing α-syn^WT^, α-syn^LP3^, or α-syn^A53T^ contained HMW insoluble α-syn species that were trapped in the stacking gel (Figure 1C). An α-syn smear was also seen at ∼ 64 kDa, which likely represent the soluble low molecular weight (LMW) α-syn conformers (Gould et al., 2014). Quantification analysis of the ratio of HMW insoluble α-syn species to LMW soluble α-syn species indicated the extent of the different α-syn species in α-syn^WT^, α-syn^LP3^, and α-syn^A53T^ (Supplementary Figure S1D). Since α-syn^LP3^ and α-syn^A53T^ showed increased ratio of HMW/LMW α-syn (Supplementary Figure S1D), and inclusion bodies similar to LB and Lewy neurites (LNs) have previously been observed in α-syn expressing *Drosophila* adult brains (Feany and Bender, 2000), we propose that the α-syn accumulations we observe within larval segmental nerves are likely insoluble forms of α-syn.

Previous work showed decreased levels of molecular motors kinesin and dynein in PD patient brains and in brains from a rat model of PD (Utton et al., 2005; Chung et al., 2009), indicating that the axonal transport pathway is likely compromised during PD. One prediction for such decreases in molecular motors is that α-syn axonal accumulations may sequester motors to these blockages thereby reducing motors from other cargoes. Indeed, expression of α-syn disrupted the axonal transport of synaptic vesicles, as assayed by the synaptic vesicle marker cysteine string protein (CSP) (Gunawardena and Goldstein, 2001; Gunawardena et al., 2013; Dolma et al., 2014). CSPs containing axonal blockages were observed in larvae expressing α-syn^WT^, in contrast to WT controls, which showed smooth CSP staining (Supplementary Figure S2A). Larvae expressing excess α-syn (α-syn^LP3^) or the fPD α-syn^A53T^ also showed CSP blocks (Supplementary Figure S2A). Quantification analysis indicated that the number of CSP blocks in larvae expressing α-syn^LP3^ or α-syn^A53T^ was significantly higher than those seen in larvae expressing α-syn^WT^ (Supplementary Figure S2B, *p* < 0.01). α-Syn^WT^-EGFP expressing larval nerves contained CSP blocks that co-localized with α-syn (Supplementary Figure S2C). Bruchpilot (BRP), an active zone protein (Kittel et al., 2006; Wagh et al., 2006; Wairkar et al., 2013) also co-localized with α-syn accumulations (Supplementary Figure S2C). Kinesin-1 assayed using an antibody against KLC was also present within α-syn accumulations (Supplementary Figure S2C). Further, the bi-directional motility of α-syn-EGFP was perturbed with 50% genetic reduction of both kinesin-1 (kinesin light chain, klc^–/+^) or dynein (dynein light chain, roblk−/+) (Supplementary Figure S3A). Note that many α-syn-EGFP-containing accumulations were observed in movie montages from larvae expressing α-syn-EGFP in the context of klc^–/+^ or roblk^–/+^. While kymographs depict the bi-directional movement of α-syn-EGFP, many stalled α-syn-EGFP tracks are seen with 50% genetic reduction of motors. Co-IP analysis done using α-syn membrane fractions shows that both kinesin (KIF5C) and dynein (DIC) are associated with α-syn (Supplementary Figure S3B). Further, the α-syn-mediated axonal transport defects we observe are not due to defects in microtubules (MTs), as the axonal MT tracks were intact in larvae expressing α-syn^WT^-EGFP as observed with the acetylated tubulin antibody (Supplementary Figure S2D). Taken together, our observations suggest that while α-syn likely associates with molecular motors for it motility within axons, excess or mutant α-syn disrupts axonal transport by sequestering motors and other synaptic cargo to α-syn accumulations.

### Disruption of α-Syn-Mediated Axonal Transport Causes Synaptic Morphological Defects in *Drosophila* NMJs

**FIGURE 2:**
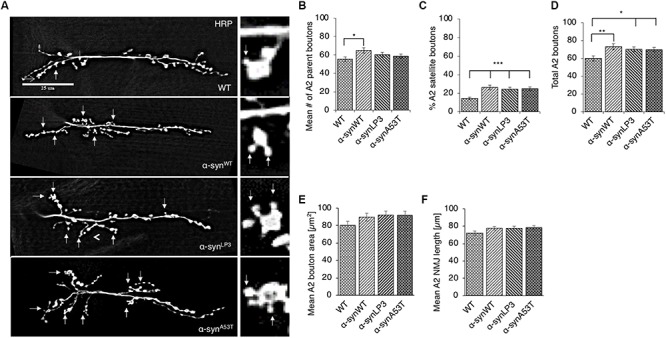
Excess human α-syn causes synaptic morphological defects in *Drosophila* NMJs. **(A)** Larval segment A2 of muscle 6/7 from wild-type (WT), α-syn^WT^, α-syn^LP3^, or α-syn^A53T^ stained with the pre-synaptic marker horseradish peroxidase (HRP). Inset of enlarged images shows a single synaptic bouton with satellite boutons budding off (arrows). **(B)** Quantitative analysis of the mean number of parent bouton in α-syn^WT^ expressing larvae shows a significant increase (*p* < 0.05) compared to WT. Expression of α-syn^LP3^ and α-syn^A53T^ shows no difference in the mean number of parent boutons. **(C)** Quantitative analysis of the percent (%) of satellite boutons in α-syn^WT^, α-syn^LP3^, and α-syn^A53T^ larvae shows a significant increase compared to WT (*p* < 0.001). **(D)** Quantification of the total number of boutons, which include both parent and satellite boutons, shows a significant increase in α-syn^WT^ (*p* < 0.01), α-syn^LP3^ (*p* < 0.05), and α-syn^A53T^ (*p* < 0.05) larvae compared to WT larvae. **(E)** Mean bouton area and **(F)** NMJs length from α-syn^WT^, α-syn^LP3^, α-syn^A53T^, or α-syn^Δ71–82^ show no differences compared to the WT control. Quantitative analysis represents mean ± SEM. ^∗^*p* < 0.05, ^∗∗^*p* < 0.01, ^∗∗∗^*p* < 0.001, *n* = 15. Bar = 25 μm.

Perturbation of axonal transport is proposed to sequester proteins that are essential for growth, function, and maintenance of synapses (Tateno et al., 2009; Goellner and Aberle, 2012; Maday et al., 2014; Sorbara et al., 2014). We previously showed that defects in axonal transport induced by two other neurodegenerative disease proteins cause synaptic morphological defects at the *Drosophila* neuromuscular junctions (NMJs) (Kang et al., 2014). Since we found that excess α-syn sequestered synaptic proteins CSP and BRP, and molecular motors within α-syn-containing accumulations (Supplementary Figure S2C), we next tested the proposal that depletion of these proteins by excess α-syn causes synaptic morphology defects. For this, we examined NMJs at muscle 6/7 in segments A2 from larvae expressing α-syn^WT^, α-syn^LP3^, or α-syn^A53T^ with the presynaptic marker, horseradish peroxidase (HRP) (Figure 2A). The well-characterized *Drosophila* NMJ is arranged in a series of synaptic boutons, called parent boutons, resembling beads on a string connected by a short neuritis (Zito et al., 1999). Loss of function mutations in motor proteins, proteins involved in axonal transport or signaling pathways that regulate synaptic growth cause the formation of “satellite” boutons, which are small ectopic boutons that emerge from the primary bouton (Zito et al., 1999; Dickman et al., 2006; O’Connor-Giles et al., 2008; Kang et al., 2014). Larvae expressing α-syn^WT^, α-syn^LP3^, or α-syn^A53T^ show an increased percentage (%) of satellite boutons at their NMJs compared to WT larval NMJs (Figure 2C, *p* < 0.001). The total number of boutons, which include satellite and parent boutons, was also significantly increased in larvae expressing α-syn^WT^ (*p* < 0.01), α-syn^LP3^ (*p* < 0.05), or α-syn^A53T^ (*p* < 0.05) compared to WT (Figures 2B–D). Likewise, NMJs from segments A3 (Supplementary Figure S4) also showed significant increases in the% of satellite boutons (Supplementary Figure S4C, α-syn^WT^, α-syn^LP3^, or α-syn^A53T^, *p* < 0.05) and in the total number of boutons (Supplementary Figure S4D, α-syn^WT^, *p* < 0.01; α-syn^LP3^, *p* < 0.05; α-syn^A53T^, *p* < 0.05) compared to WT. Note that there were no significant differences seen in the percentage of satellite boutons or the total number of boutons between α-syn^WT^ expressing larvae and α-syn^LP3^ or α-syn^A53T^ expressing larvae (Figure 2 and Supplementary Figure S4). No significant changes were seen in the synaptic bouton area or in the NMJ length between WT larvae and larvae expressing α-syn^WT^, α-syn^LP3^, or α-syn^A53T^ (Figures 2E,F and Supplementary Figures S4E,F). Collectively, our observations indicate that excess or mutant α-syn affect synaptic morphology.

Previous work showed that α-syn expression decreased synaptic vesicle density and the levels of synaptic protein such as synapsin in mouse brains (Nemani et al., 2010). To test the proposal that decreased transport of synaptic proteins contribute to the α-syn-mediated morphological abnormalities we observe in larval synapses, we examined the intensity of endogenous CSP, a protein which functions in synaptic growth (Fernández-Chacón et al., 2004; Zinsmaier, 2010), at muscle 6/7 of segments A2 in larvae expressing α-syn^WT^, α-syn^LP3^, or α-syn^A53T^ (Supplementary Figure S5A). Intriguingly, significant reductions in the intensity of CSP in larval NMJs expressing α-syn^WT^, α-syn^LP3^, or α-syn^A53T^ were seen compared to WT (Supplementary Figure S5B, *p* < 0.05). We also evaluated the level of BRP, the active zone protein that is suggested to be important for neurotransmitter release at the *Drosophila* synapse (Kittel et al., 2006; Wagh et al., 2006; Wairkar et al., 2013) at muscle 4 segment A2. Since *Drosophila* larval NMJs at muscle 4 are relatively small in size compared to muscle 6/7 NMJs, we examined the entire NMJ with a higher objective lens (60×). We found that expression of α-syn at higher levels (α-syn^LP3^) or expression of fPD mutant A53T (α-syn^A53T^) significantly reduced the number of BRP puncta or BRP intensity at the NMJ compared to WT (Supplementary Figures S5C–E, BRP puncta, *p* < 0.01; BRP intensity, *p* < 0.05). Although larval NMJs expressing α-syn^WT^ also showed reduced levels of BRP puncta and intensity, these levels were not statistically significant from what was seen in WT (Supplementary Figures S5C–E). Since both CSP and BRP were present within α-syn-containing axonal accumulations (Supplementary Figure S2C), these observations suggest that the α-syn-mediated synaptic morphological abnormalities could arise due to perturbation of the axonal transport of essential synaptic proteins.

### Disruption of Axonal Transport Mediated by α-Syn Impairs Larval Locomotion

**FIGURE 3:**
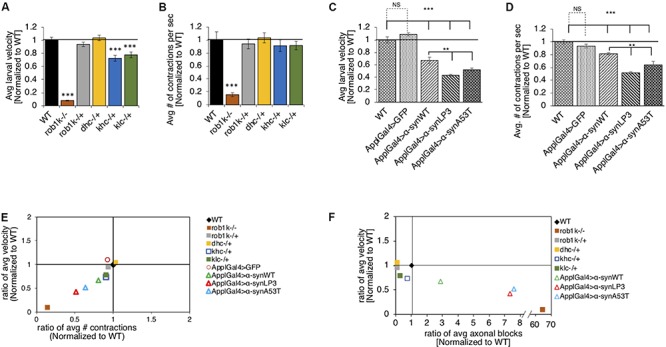
Excess human α-syn causes larval locomotion defects, similar to what was observed in molecular motor protein mutant larvae. **(A)** Quantification analysis of the average larval crawling velocity (normalized to WT) from larvae with a complete loss of dynein (robI^–/+^), or a 50% reduction of kinesin (khc^–/+^ or klc^–/+^) or dynein (robI^*k*–/+^ or dhc^–/+^) normalized to WT control. The larval crawling velocity of larvae carrying a homozygous dynein (robl^*k*–/–^, *p* < 0.001) mutation was significantly reduced compared to WT, while heterozygous dynein larvae (robl^*k*–/–^ or dhc^–/+^) were similar to WT. Larvae with 50% reduction of kinesin-1 (khc^–/+^ or klc^–/+^) show a significant decrease in larval crawling velocity compared to WT (*p* < 0.001). **(B)** The average number of peristaltic contractions during 60 s (normalized to WT) for motor protein mutants. Note that only homozygous dynein robl^*k*–/–^ (*p* < 0.001) larvae show significant decreases in contractions. **(C)** Quantification of the average crawling velocity (normalized to WT) for larvae expressing α-syn^WT^, α-syn^LP3^, or α-syn^A53T^ are significantly reduced compared to WT or GFP alone (*p* < 0.001). The average velocity of larvae expressing α-syn^LP3^ or α-syn^A53T^ was significantly reduce compared to α-syn^WT^ larvae (*p* < 0.01). **(D)** Quantitative analysis of the average number (#) of contractions in 60 s (normalized to WT) for larvae expressing α-syn^WT^, α-syn^LP3^, or α-syn^A53T^ indicates significant decreases in contractions compared to WT or GFP alone (*p* < 0.001). A significant reduction was also observed in the average larval contractions of larvae expressing α-syn^LP3^ or α-syn^A53T^ compared to α-syn^WT^ (*p* < 0.01). **(E)** The ratio of the average contractions (*X*-axis) plotted against the ratio of the average velocities (*Y*-axis) (WT normalized to 1) for motor mutants and α-syn expressing larvae. Note that as contractions decease so does the larval velocity. **(F)** The ratio of the average number of axonal blocks (*X*-axis) plotted against the ratio of the average larval velocity (*Y*-axis) for motor protein mutants and α-syn-expressing larvae. Note that as the number of axonal blocks increases, the larval velocities decrease. Quantitative analysis represents the mean ± SEM. ^∗∗^*p* < 0.01, ^∗∗∗^*p* < 0.001, *n* = 10.

Previous work has shown that expression of α-syn in *Drosophila* adult flies causes climbing defects (Feany and Bender, 2000). To test the proposal that such behavioral defects are due to perturbations in long distance axonal transport, we evaluated larval crawling velocities and peristaltic body wall contraction in α-syn^WT^, α-syn^LP3^, or α-syn^A53T^ expressing larvae. The *Drosophila* central pattern generator (CPG), a neural network, is thought to control the coordination of peristaltic movement of larval body muscles, which result in crawling (Marder and Calabrese, 1996; Marder and Bucher, 2001; Fox et al., 2006). A full peristaltic contraction of the body wall starts at the posterior end of the larvae and propagate in a wave-like manner to the anterior end of the larvae, terminating at the mouth hooks (Fox et al., 2006). Since larval crawling speeds increase with the frequency of contractions, speed, body muscle contractions, and the distance traveled are thought to be correlated (Berrigan and Pepin, 1995; Aleman-Meza et al., 2015). To test the proposal that defects in axonal transport contributes to larval locomotion, we first evaluated the crawling velocity and body wall contraction of larvae with complete loss of function (homozygous) or 50% reduction (heterozygous) of molecular motor proteins. *Drosophila* larvae with a complete loss of kinesin-1 (kinesin heavy chain, khc^–/–^ or kinesin light chain, klc^–/–^) or dynein (dynein light chain, rob1k^–/–^ or dynein heavy chain, dhc^–/–^) display a neuromuscular tail “flip” phenotype and paralysis of the posterior region (Saxton et al., 1991; Gindhart et al., 1998; Bowman et al., 1999; Martin et al., 1999). In contrast, larvae with 50% reduction in kinesin-1 (klc^–/+^ or khc^–/–^) or dynein (rob1k^–/+^ or dhc^–/+^) appear normal with no noticeable crawling defect, and their segmental nerves stain smoothly with CSP (Saxton et al., 1991; Gindhart et al., 1998; Bowman et al., 1999; Martin et al., 1999; Gunawardena and Goldstein, 2001). We found that rob1k^–/–^ larvae display significant decreases in crawling velocities (*p* < 0.001) and body wall contractions (*p* < 0.001), while rob1k^–/+^ or dhc^–/+^ larvae were comparable to WT (Figures 3A,B). Interestingly, khc^–/+^ or klc^–^/^+^ larvae display significantly reduced larval crawling velocities (*p* < 0.001), but no changes were seen in body wall contractions (Figures 3A,B). Therefore, disruption of long-distance transport within axons influences larval locomotion behaviors.

It is possible that the axonal blockages we observe in α-syn expressing larval segmental nerves (Figure 1) also contribute to larval locomotion defects. Indeed, larvae expressing α-syn^WT^, α-syn^LP3^, or α-syn^A53T^ in all neurons, using the pan neuronal driver, APPL-GAL4, showed significant decreases in larval crawling velocities compared to WT (Figure 3C, *p* < 0.001). While we previously showed that expression of just any protein does not cause axonal blockages (Gunawardena and Goldstein, 2001; Gunawardena et al., 2003), we also examined whether crawling defects were specific to the expression of α-syn by examining the crawling behavior of larvae expressing GFP alone. Indeed, the crawling defects we observe were not due to the expression of just any protein in all larval neurons, but rather specific to α-syn expression, as larvae expressing GFP alone had no effect and were comparable to WT larvae (Figure 3C). Strikingly, larvae expressing α-syn^LP3^ or the fPD mutant α-syn^A53T^ showed significantly lower crawling velocities compared to α-syn^WT^ larvae (Figure 3C, *p* < 0.001). Since larval locomotion velocities are correlated to larval body wall contractions, we next measured the number of full body peristaltic contractions performed by a larva in 60 s. Strikingly, pan neuronal expression of α-syn^WT^, α-syn^LP3^, or α-syn^A53T^ resulted in significant decreases in larval body wall contractions compared to WT or to larvae expressing GFP alone (Figure 3D, *p* < 0.001). Expression of α-syn^LP3^ or the fPD mutant α-syn^A53T^ also decreased body wall contractions and these decreases were significant compared to larvae expressing α-syn^WT^ (Figure 3D, *p* < 0.001). Similarly, expression of α-syn^WT^, α-syn^LP3^, or α-syn^A53T^ in dopaminergic neurons using the TH-GAL4 driver also showed significant decreases in larval crawling velocities and body wall contractions (Supplementary Figures S6A,B, *p* < 0.001). To further evaluate larval locomotion behaviors, we plotted larval crawling velocities against body wall contractions (Figure 3E). Larvae with complete loss of dynein function (rob1k^–/–^) were at the bottom of the quadrant showing the greatest correlation with decreased crawling velocities and decreased contraction, while larvae with 50% reduction of motors showed the least correlation and were at the top of the quadrant (Figure 3E). Interestingly, larvae expressing α-syn were at the middle of the quadrant with larvae expressing excess α-syn showing a correlation that was closer to loss of dynein function larvae. To evaluate how the extent of axonal blockages correlates to larval locomotion, we next plotted the ratio of the average number of axonal blocks against the ratio of the average larval crawling velocities. Strikingly, larvae with complete loss of dynein function, which contain many axonal blocks within their larval segmental nerves (Gunawardena and Goldstein, 2001), were at the bottom of the quadrant, while α-syn expressing larvae (α-syn^LP3^ or α-syn^A53T^) were at the middle of the quadrant. Note that larvae that were heterozygous for motor protein mutants were in a different quadrant and were closer to WT larvae (Figure 3F). Taken together, our observations suggest that larval locomotion behaviors are likely to be directly influenced by defects in axonal transport.

### The Axonal Entry and Transport of α-Syn Is Dependent on Amino Acid 71-82

**FIGURE 4:**
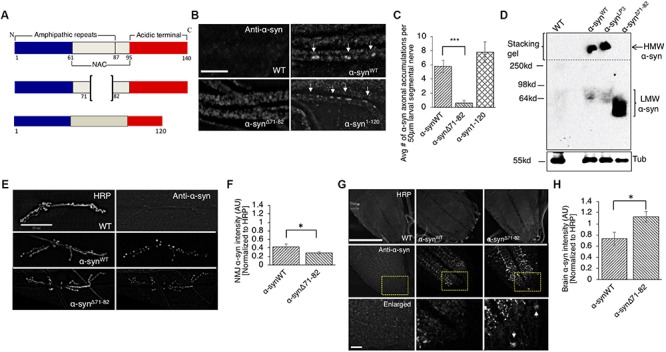
The NAC region is required for the axonal transport of α-syn to synapses. **(A)** Schematic representation of full-length α-syn (140 amino acids), α-syn with a deletion of region 71-82 in the non-amyloidal component (NAC, α-syn^Δ71–82^), and α-syn lacking the C-terminal region (1-120, α-syn^1–120^) (not drawn to scale). **(B)** α-syn antibody staining within α-syn^Δ71–82^ expressing larval segmental nerves appear mostly diffused while α-syn^WT^ or α-syn^1–120^ larval nerves show axonal accumulations (arrow, bar = 25 μm). **(C)** Quantification of the average number of α-syn accumulations within α-syn^Δ71–82^ larval segmental nerves indicates significant decreases as compared to α-syn^WT^ larval nerves (*p* < 0.001), while the extent of α-syn accumulations in α-syn^1–120^ larval segmental nerves is similar to α-syn^WT^ (*n* = 7). **(D)** Native non-denaturing gel electrophoresis and Western blot analysis indicate that α-syn^Δ71–82^ does not form higher molecular weight HMW) α-syn species in flies. Note a low molecular weight (LMW) smear around 60–64 kDa. Tubulin is used as a loading control. **(E)** α-syn and HRP staining in WT, α-syn^WT^, and α-syn^Δ71–82^ larval NMJs at segment A2 of muscle 6/7 (bar = 25 μm). **(F)** Quantitative analysis indicates a significant decrease in α-syn intensity (normalized to HRP) at α-syn^Δ71–82^ larval NMJs compared to α-syn^WT^ larval NMJs (*p* < 0.05, *n* = 10). **(G)** α-Syn and HRP staining within WT, α-syn^WT^, and α-syn^Δ71–82^ larval brains. Bottom panel represents an enlarge portion of the brain (inset). Note the accumulation of α-syn within the cell body within α-syn^Δ71–82^ expressing larvae (arrow). **(H)** Quantification of α-syn intensity (normalized to HRP) within the brain of α-syn^Δ71–82^ larvae indicates a significant increase in α-syn compared to α-syn^WT^ larval brains (*p* < 0.05, *n* = 5). The quantitative analysis represents the mean ± SEM. ^∗^*p* < 0.05, ^∗∗∗^*p* < 0.001. AU = arbitrary units.

The NAC region of α-syn, specifically amino acids 71-82, is thought to be essential for α-syn aggregation (Bodles et al., 2001; Giasson et al., 2001). It was proposed that this region undergoes a conformational change from random coils to a β-sheet structure, which can form cylindrical β-sheets and amyloid-β-like fibrils (Bellucci et al., 2012; Deleersnijder et al., 2013). Since a previous study showed that expression of human α-syn lacking amino acid 71-82 in *Drosophila* prevented DA neuronal loss induced by excess α-syn (Periquet et al., 2007), we tested the proposal that deletion of amino acid 71-82 (Figure 4A) also modulates axonal transport defects induced by excess α-syn. Larvae expressing α-syn^Δ71–82^ showed little to no α-syn accumulations and α-syn appeared diffused within larval segmental nerves (Figures 4B,C, *p* < 0.001). These effects were not due to changes in the level of protein expression since Western blot analysis show that α-syn^Δ71–82^ is expressed at similar levels to α-syn^WT^ (Supplementary Figures S1A,B). In addition, α-syn^Δ71–82^ expressing larval nerves showed little to no CSP blocks and larval nerves were smoothly stained similar to what was seen in WT larvae (Supplementary Figure S7A). Quantification analysis indicated that α-syn^Δ71–82^ expressing larvae were similar to WT control larvae, but were significantly different to what was observed in α-syn^WT^ larvae (*p* < 0.01) (Supplementary Figure S7B). However, expression of α-syn lacking a portion of the C-terminal region, α-syn^1–120^ contained α-syn and CSP blockages similar to the extent seen in α-syn^WT^ (Figures 4A–C and Supplementary Figures S7A,B).

One proposal for the lack of α-syn accumulations within larval nerves is that the NAC region is involved in influencing the transport of α-syn within larval axons. To evaluate this proposal, we examined and quantified the intensity of α-syn at the NMJ in larvae expressing α-syn^Δ71–82^. We found significant reductions in α-syn intensities at NMJs of larvae expressing α-syn^Δ71–82^ compared to α-syn^WT^ (Figures 4E,F, *p* < 0.05). Strikingly, α-syn intensities in larval brains (where the cell bodies are located) from larvae expressing α-syn^Δ71–82^ were significantly higher compared to α-syn^WT^ expressing larval brains (Figures 4G,H, *p* < 0.05). These observations suggest that the 71-82 amino acid region of α-syn is important for its axonal entry and for its transport within the axon.

**FIGURE 5:**
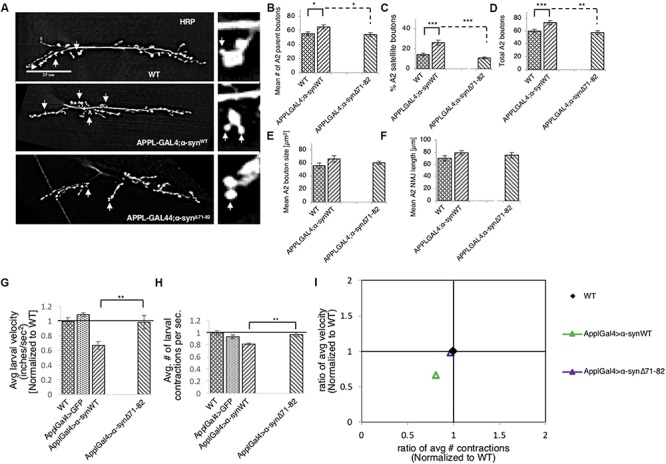
Deletion of the NAC region rescues α-syn-mediated synaptic morphological defects and larval locomotion defects. **(A)** Larval segment A2 of muscle 6/7 from wild-type (WT), α-syn^WT^, or α-syn^Δ71–82^ stained with the pre-synaptic marker horseradish peroxidase (HRP). Inset of enlarged images shows a single synaptic bouton with satellite boutons budding off (arrows). **(B)** Quantitative analysis of the mean number of parent bouton in α-syn^Δ71–82^ expressing larvae shows a significant decrease (*p* < 0.05) compared to α-syn^WT^. Note that α-syn^Δ71–82^ is comparable to WT. **(C)** Quantitative analysis of the percent (%) of satellite boutons in α-syn^Δ71–82^ larvae shows a significant decrease compared to α-syn^Δ71–82^ (*p* < 0.001) similar to WT. **(D)** Quantification of the total number of boutons, which include both parent and satellite boutons, shows a significant decrease in α-syn^Δ71–82^ (*p* < 0.01) compared to α-syn^Δ71–82^ and similar to WT. **(E)** Mean bouton area and **(F)** NMJs length from α-syn^WT^ or α-syn^Δ71–82^ show no differences compared to the WT control. Quantitative analysis represents mean ± SEM. ^∗^*p* < 0.05, ^∗∗^*p* < 0.01, ^∗∗∗^*p* < 0.001, *n* = 15. Bar = 25 μm. **(G)** Quantification of the average crawling velocity (normalized to WT) for larvae expressing α-syn^WT^ or α-syn^Δ71–82^ compared to WT. The average velocity of larvae expressing α-syn^Δ71–82^ was significantly increased compared to α-syn^WT^ larvae (*p* < 0.01) and was comparable to WT. **(H)** Quantitative analysis of the average number (#) of contractions in 60 s (normalized to WT) for larvae expressing α-syn^Δ71–82^ indicates a significant increase in contractions compared to α-syn^WT^ (*p* < 0.01) and was similar to WT. **(I)** The ratio of the average contractions (*X*-axis) plotted against the ratio of the average velocities (*Y*-axis) (WT normalized to 1) for α- α-syn^WT^ and α-syn^Δ71–82^ expressing larvae. Note that α-syn^Δ71–82^ larvae are similar to WT larvae and distinct from α-syn^WT^ larvae. Quantitative analysis represents the mean ± SEM. ^∗∗^*p* < 0.01, *n* = 10.

To test how the NAC region of α-syn contributes to synaptic morphology, we examined NMJs from larvae expressing α-syn^Δ71–82^ and found that these NMJs were comparable to NMJs from WT larvae. When comparing NMJs from larvae expressing α-syn^Δ71–82^ to larvae expressing α-syn^WT^, significant decreases were seen in the average number of parental boutons and in the% of satellite boutons which correspond to the significant decrease seen in the total amount of boutons (Figures 5A–F and Supplementary Figure S8). These results are consistent with the observation that no significant changes to CSP or BRP intensities were observed in NMJs from larvae expressing α-syn^Δ71–82^ in contrast to NMJs from larvae expressing α-syn^WT^ (Supplementary Figures S9A–E). Larval crawling behaviors in larvae expressing α-syn^Δ71–82^ were also comparable to the crawling behaviors seen in WT larvae (Figures 5G,H and Supplementary Figure S10). Note that a significant increase in the average larval velocity and in the average number of contractions was seen in larvae expressing α-syn^Δ71–82^ compared to larvae expressing α-syn^WT^. When we plot the larval crawling velocities against body wall contractions, both α-syn^Δ71–82^ larvae and WT larvae fall at the intersection of the quadrants (Figure 5I). Therefore, NAC-mediated axonal α-syn accumulations directly perturb the axonal transport of synaptic proteins contributing to the synaptic morphological defects and larval crawling defects we observe.

To test the proposal that the α-syn-mediated axonal transport defects, synaptic morphological defects and larval crawling defects are due to α-syn aggregation, we evaluated whether α-syn^Δ71–82^ expressing flies formed HMW insoluble α-syn species as assayed by native none-denaturing gel electrophoresis and Western blot analysis (Figure 4D). Note that only soluble LMW soluble α-syn species was detected in α-syn^Δ71–82^ expressing flies in contrast to α-syn^WT^ expressing flies. Quantification analysis revealed a low HMW/LMW ratio α-syn for α-syn^Δ71–82^ in contrast to α-syn^LP3^ (Supplementary Figure S11). Taken together, these observations suggest that the α-syn accumulations we observe in larvae expressing α-syn^WT^ or α-syn^LP3^ are likely dependent on amino acids 71-82 of α-syn.

### The NAC Region of α-Syn Associates With Membranes for Its Axonal Transport

**FIGURE 6:**
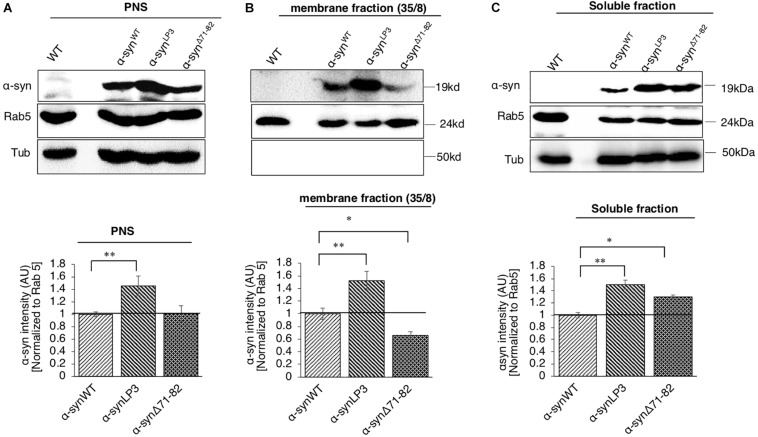
α-Syn binds membranes *via* the NAC region. **(A)** Postnuclear supernatant (PNS) shows the extent of α-syn expression in larvae. Quantification analysis of the PNS fraction shows the expression of α-syn in larval brains. Note that α-syn^LP3^ larval brains contain a higher amount of α-syn compared to α-syn^WT^ (*p* < 0.01). The level of α-syn in α-syn^Δ71–82^ larval brains was similar to what was seen in α-syn^WT^ larval brains. **(B)** Subcellular fractionation of larval brains on sucrose step gradients indicates that α-syn is present in the membrane fraction and in the soluble fraction. Quantification analysis of the light membrane fraction (35/8) indicates a significantly high level of α-syn with membranes in α-syn^LP3^ larval brains (*p* < 0.01). Note that a significant decrease of membrane bound α-syn is seen in α-syn^Δ71–82^ larval brains compared to α-syn^WT^ (*p* < 0.05). **(C)** Quantification of the soluble fraction shows an increase level of α-syn levels in α-syn^LP3^ (*p* < 0.01) brains compared to α-syn^WT^ brains. Note that the level of α-syn levels in α-syn^Δ71–82^ brains was increased comparable to α-syn^WT^ brains (*p* < 0.05). Antibodies against α-syn, Rab5 (for membranes), and Tubulin (loading control) were used. The ratio of relative intensity of α-syn was normalized to the intensity of Rab5 positive for membranes and then normalized to α-syn^WT^. The level of intensity in α-syn^WT^ was set at 1. The ratio of the relative intensity of proteins normalized to Rab5 is shown from three independent experiments (*n* = 3, AU = arbitrary units). The quantitative analysis represents mean ± SEM. ^∗^*p* < 0.05, ^∗∗^*p* < 0.01.

One reason for the decrease in α-syn at the NMJs of α-syn^Δ71–82^ larvae is the involvement of this region in binding membranes. To test this prediction, we isolated and biochemically analyzed α-syn on membranes using sucrose gradients from larval brains expressing α-syn^WT^, α-syn^LP3^, and α-syn^Δ71–82^. α-Syn^WT^, α-syn^LP3^, and α-syn^Δ71–82^ are expressed in larval brains as seen in the post nuclear supernatant (PNS) (Figure 6A). We found that α-syn is present in the 35/8 membrane fraction and in the soluble fraction (Figures 6B,C). Surprisingly, α-syn^LP3^ was significantly increased the amount of α-syn in the 35/8 membrane fraction compared to α-syn^WT^ (Figure 6B, *p* < 0.01). In contrast, α-syn^Δ71–82^ was significantly decreased in the 35/8 membrane fraction compared to α-syn^WT^ (Figure 5A, *p* < 0.05). The reductions seen in the membrane fraction for α-syn^Δ71–82^ correlated with increases in the soluble fraction (Figure 6C, *p* < 0.05). Therefore, our observations suggest that amino acids 71-82 in α-syn are likely important for membrane association. Perhaps the decrease in α-syn intensity we observe in α-syn^Δ71–82^ expressing larval NMJs (Figures 4E,F) can be attributed to decreases in the axonal entry of the membrane bound fraction of α-syn and its transport within axons.

### Expression of α-Syn^Δ71–82^ Rescues α-Syn-Mediated Axonal Accumulations, Synaptic Morphology Defects and Decreases the Formation of Higher Molecular Weight α-Syn Species

**FIGURE 7:**
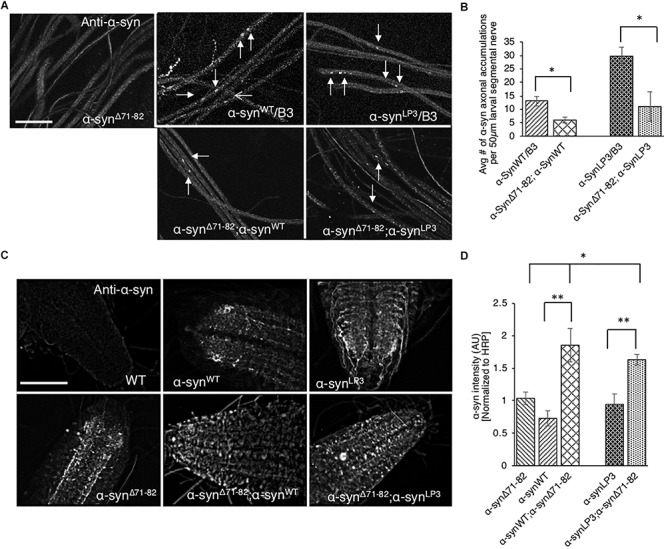
Expression of α-syn lacking the NAC region suppresses α-syn accumulations within larval nerves and increases α-syn in larval brains. **(A)** Larval segmental nerves expressing α-syn^Δ71–82^ with α-syn^WT^ or α-syn^LP3^ stained with the α-syn antibody. The α-syn^WT^/B3 and α-syn^LP3^/B3 segmental nerves are siblings from each genetic cross and show α-syn accumulations (arrows). Note that the amounts of α-syn accumulations in larval segmental nerves from larvae co-expressing α-syn^Δ71–82^;α-syn^WT^ or α-syn^LP3^;α-syn^Δ71–82^ are reduced compared to sibling larvae expressing α-syn^WT^ or α-syn^LP3^ alone (arrows, bar = 10 μm). **(B)** Quantitative analysis indicates significant decreases in α-syn accumulations in larval segmental nerves co-expressing α-syn^Δ71–82^;α-syn^WT^ or α-syn^LP3^;α-syn^Δ71–82^ compared to larvae expressing α-syn^WT^/B3 or α-syn^LP3^/B3 (*p* < 0.05, *n* = 6). The quantitative analysis represents mean ± SEM. ^∗^*p* < 0.05. **(C)** α-Syn staining in larval brains from α-syn^Δ71–82^;α-syn^WT^ or α-syn^Δ71–82^;α-syn^LP3^ compared to α-syn^WT^, α-syn^LP3^ or α-syn^Δ71–82^ alone. **(D)** Quantification of α-syn intensity (normalized to HRP) within larval brains from co-expressing α-syn^Δ71–82^;α-syn^WT^ or α-syn^Δ71–82^;α-syn^LP3^ compared to larvae expressing α-syn^WT^, α-syn^LP3^, or α-syn^Δ71–82^ alone indicates a significant increase in α-syn (^∗^*p* < 0.05, ^∗∗^*p* < 0.01, *n* = 5). The quantitative analysis represents the mean ± SEM. AU = arbitrary units.

Perhaps the axonal α-syn accumulations we observe (Figure 1A) are due to increased association of α-syn to membranes (Figure 6). Indeed, increasing the expression of α-syn (α-syn^LP3^) in larval nerves caused an increased amount of α-syn to bind to membranes compared to larvae expressing α-syn^WT^ (Figure 6). Previous *in vitro* studies proposed that α-syn can bind vesicles or membranes as dimers, and that a hydrophobic interaction between the α-syn NAC region and the lipid membrane promotes the formation of α-syn dimers due to a conformational change (Colebc et al., 2002; Jo et al., 2002). α-Syn dimers were also suggested to act as intermediates for α-syn aggregation (Hashimoto et al., 1998; Wood et al., 1999; Giráldez-Pérez et al., 2014). Therefore, to test the proposal that the NAC region is involved in promoting axonal α-syn accumulations *in vivo*, we co-expressed α-syn^Δ71–82^ with excess α-syn. Intriguingly, larvae co-expressing α-syn^Δ71–82^ and α-syn^WT^ (α-syn^Δ71–82^;α-syn^WT^) show significantly less α-syn accumulations within larval segmental nerves compared to larvae expressing α-syn^WT^ alone (Figures 7A,B, *p* < 0.05). Larvae co-expressing α-syn^Δ71–82^ and α-syn^LP3^ (α-syn^Δ71–82^;α-syn^LP3^) also show a significant reduction in axonal α-syn accumulations compared to larvae expressing α-syn^LP3^ alone (Figures 7A,B, *p* < 0.05). Since decreased axonal α-syn accumulations could be due to decreased axonal entry and transport of α-syn, we next evaluated the extent of α-syn intensity within larval brains and NMJs. Intriguingly, we found that larval brains from larvae co-expressing α-syn^Δ71–82^ and α-syn^WT^ or α-syn^Δ71–82^ and α-syn^LP3^ showed increased α-syn intensity normalized to HRP as compared to α-syn^WT^ or α-syn^LP^ alone (*p* < 0.001), or to α-syn^Δ71–82^ alone (*p* < 0.05, Figures 7C,D), while no significant changes to α-syn intensities were seen at these larval NMJs (Supplementary Figures S12A,B). These observations indicate that the rescue of α-syn-containing axonal accumulations seen in larvae co-expressing α-syn^Δ71–82^ with α-syn^WT^ or α-syn^LP3^ is not due to reductions in axonal entry or the axonal transport of α-syn.

**FIGURE 8:**
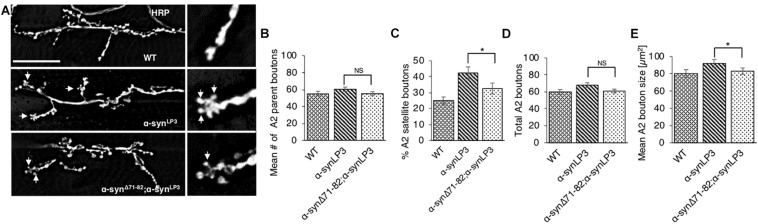
Expression of α-syn lacking the NAC region rescuesα-syn-mediated synaptic morphological defects within larval NMJs. **(A)** Larval segment A2 of muscle 6/7 from wild-type (WT) or α-syn^LP3^ larvae and larvae co-expressing α-syn^LP3^;α-syn^Δ71–82^ stained with the pre-synaptic marker horseradish peroxidase (HRP). Inset of enlarged images shows a single synaptic bouton with satellite boutons budding off (arrows). **(B)** Quantitative analysis of the mean number of parent bouton in α-syn^LP3^;α-syn^Δ71–82^ expressing larvae shows a decrease which is not significant compared to α-syn^LP3^. **(C)** Quantitative analysis of the percent (%) of satellite boutons in α-syn^LP3^;α-syn^Δ71–82^ larvae shows a significant decrease compared to α-syn^LP3^ (*p* < 0.001). **(D)** Quantification of the total number of boutons, which include both parent and satellite boutons, shows a decrease in α-syn^LP3^;α-syn^Δ71–82^ compared to α-syn^LP3^ which is similar to WT. **(E)** Mean bouton area from α-syn^LP3^;α-syn^Δ71–82^ shows a significant decrease compared to α-syn^LP3^ which is similar to the WT control. Quantitative analysis represents mean ± SEM. ^∗^*p* < 0.05, NS = not significant, *n* = 5. Bar = 25 μm.

To test the prediction that expression of α-syn^Δ71–82^ with α-syn^LP3^ restores the transport of essential synaptic proteins for the maintenance and function of NMJs, we next examined NMJs from larvae expressing α-syn^Δ71–82^ with α-syn^LP3^. We found that co-expression of α-syn^Δ71–82^ with α-syn^LP^ significantly rescued the% of satellite boutons and the average bouton size seen in α-syn^LP^ NMJs (Figures 8A–E, *p* < 0.05). These observations support the proposal that the NAC region is involved in promoting axonal α-syn accumulations disrupting the transport of essential proteins to synapses.

Since it was proposed that a hydrophobic interaction between the NAC region and lipid membranes promotes the formation of α-syn dimers (Colebc et al., 2002; Jo et al., 2002), which cause α-syn aggregation (Hashimoto et al., 1998; Wood et al., 1999; Giráldez-Pérez et al., 2014), we biochemically probed how deletion of the NAC region affects the behavior of α-syn by evaluating different species of α-syn using native none-denaturing gel electrophoresis and Western blot analysis. Obtaining sufficient sample of progeny that co-express both α-syn^WT^ or α-syn^LP3^ and α-syn^Δ71–82^ for this analysis required considerable effort as only 25% of the genotypes were α-syn^WT^;α-syn^Δ71–82^ or α-syn^LP3^;α-syn^Δ71–82^ from our genetic crosses (see the section “Materials and Methods”). While expression of α-syn^WT^ or α-syn^LP3^ alone showed HMW species of α-syn in the staking gel, co-expression of α-syn^Δ71–82^ with α-syn^WT^ or α-syn^LP3^ showed lower molecular weight (LMW) species of α-syn at 64 kDa (compare lanes 1 and 2 to lanes 5 and 6 in Supplementary Figure S13A). Quantification analysis of the ratio of HMW to LMW showed a lower HMW/LMW ratio for α-syn^Δ71–82^ alone and α-syn^WT^ or α-syn^LP3^ with α-syn^Δ71–82^ (Supplementary Figure S13B), indicating that the α-syn^Δ71–82^-mediated rescue of α-syn-containing axonal accumulations and synaptic morphological defects are perhaps due to HMW insoluble α-syn species. While further study is needed to isolate the structural details of how the NAC region facilitates α-syn protein–protein interactions on membranes, taken together our observations indicate that the NAC region plays an essential role in α-syn associations on axonal membranes and its transport within axons under physiological conditions.

## Discussion

Our study provides important insight into the *in vivo* function for the NAC region of α-syn during its transport within axons. Specifically, we found that the NAC region is essential for the movement of α-syn within larval axons and is required for membrane association of α-syn, perhaps *via* an NAC-mediated α-syn protein–protein interaction mechanism. Further, excess α-syn impedes the transport of synaptic vesicles causing synaptic defects and larval locomotion defects. Collectively, these observations propose a model in which too much α-syn perturbs axonal transport *via* aggregate formation, instigating synaptic and clinical pathology seen in PD (Supplementary Figure S14).

α-Syn is a pre-synaptic protein with functions implicated in synaptic vesicle trafficking and neurotransmission (Abeliovich et al., 2000; Murphy et al., 2000; Cabin et al., 2002; Inoshita and Imai, 2015). α-Syn moves bi-directionally within axons to synapses (Jensen et al., 1999; Roy et al., 2008; Yang et al., 2010), and likely binds to both kinesin-1 and dynein as see by co-IP analysis with motor proteins (Utton et al., 2005; Supplementary Figures S3A,B). Consistent with these observations, we found that α-syn is present within larval axons and is transported to larval NMJs (Figures 1, 4). Intriguingly, the deletion of the NAC region of α-syn, but not the C-terminal region decreased NMJ localization of α-syn suggesting that the NAC region is required for the normal transport of α-syn within axons (Figure 4). In agreement with our findings, work in primary hippocampal neurons has demonstrated that deletion of exon 4, which spans aa 57-102 (which includes the NAC region), but not exon 5 or 6 (a partial deletion of the C-terminal) decreased presynaptic localization of α-syn (Yang et al., 2010). Since proper transport and presynaptic localization of α-syn has been proposed to be important in regulating synaptic vesicle trafficking and exocytosis (Murphy et al., 2000; Chandra et al., 2005; Yang et al., 2010), perhaps the LB inclusions seen in PD brains which were proposed to be due to the mis-localization of α-syn (Kahle et al., 2000; Saha et al., 2004; Yang et al., 2010) are caused by defects to the normal axonal transport of α-syn.

Excess of α-syn perturbs its own movement within axons, causes α-syn accumulations within axons, disrupts the movement of synaptic vesicle proteins, and decreases their levels at the synapse (Figures 1, 4 and Supplementary Figures S2, S5). Expression of excess α-syn exaggerated these effects. These phenotypes were also seen in larvae expressing the fPD mutation A53T which alters the structure of α-syn. Since triplication and duplication of α-syn genes also cause PD (Singleton et al., 2003; Ibáñez et al., 2004), we propose that excess levels of α-syn as well as structurally defective α-syn can directly impede the axonal transport of α-syn, and other important synaptic components to the synapse.

There are at least two possible mechanisms by which excess or mutant α-syn could disrupt normal axonal transport. First, increased levels of α-syn could sequester motor proteins away from other synaptic cargo, thereby inhibiting normal axonal transport. Indeed, previous work has shown decreased levels of both kinesin and dynein motor proteins in PD patient brains and in brains from a rat model of PD (Chung et al., 2009; Chu et al., 2012). LB inclusions were reported to contain synaptic proteins such as synphilin and synaptophysin (Katsuse et al., 2003). Consistent with this, we find that while α-syn accumulations are likely to contain phosphorylated α-syn, they also contain motors and synaptic proteins (Supplementary Figure S2C). In α-syn transgenic mice, the rate of axonal transport of α-syn slowed with age (Li et al., 2003), together with axonal swellings or accumulation of neurofilaments (Kahle et al., 2000; Lee et al., 2002). Direct application of preformed α-syn fibrils to neurons *in vitro* impeded the axonal transport of endosomes (Volpicelli-Daley et al., 2014), and increased α-syn dosage, resulting in α-syn oligomerization, which in turn perturbed the axonal transport of mitochondria in human neurons (Prots et al., 2018). Alternatively, structural changes or aberrant folding of α-syn could interfere with α-syn binding to motors causing defects in axonal transport. Indeed, the fPD mutation A53T, which is thought to alter the normal structure of α-syn (Sahay et al., 2015), shows transport defects similar to defects seen in excess α-syn (Figure 1 and Supplementary Figure S2). FRET studies indicate that the A53T mutation alters α-syn association with the α-syn-interacting protein synphilin-1 (Kawamata et al., 2001). It is also possible that aberrant dimerization or oligomerization under conditions of excess α-syn seen in duplication/triplication PD or mutant α-syn seen in fPD (Breydo et al., 2012) causes conformational changes in α-syn associations with membranes or α-syn interactions with other proteins. Intriguingly, increases in the concentration of α-syn have been suggested to shift the structure of α-syn to a partially folded conformation, which represents a key intermediate in the fibrillation pathway (Uversky, 2007).

The second possible mechanism for how α-syn disrupts axonal transport could involve the impairment of structure and stability of MT within axons by excess α-syn, as *in vitro* studies have shown that α-syn can interact with tubulin (Zhou et al., 2010) and the MT-associated protein (MAPT) (Benussi et al., 2005; Roy and Jackson, 2014). Indeed, excess α-syn induced tubulin polymerization defects (Prots et al., 2013). α-Syn fPD mutations increased tau phosphorylation (Wills et al., 2011) and tau hyperphosphorylation (Kanaan et al., 2011). Therefore, since transport within axons depend on an intact MT, defects in tubulin polymerization and or aberrant interactions with MAPT mediated by excess α-syn could also disrupt long distance transport. Although our observations suggest that this proposal is unlikely given that intact acetylated tubulin-positive MT tracks were observed in larvae expressing α-synWT-EGFP (Supplementary Figure S2D), further investigations will be needed to test this proposal. While these two mechanisms may not be mutually exclusive, perhaps a threshold could exist where vast increases in α-syn levels or changes in α-syn structure caused by aberrant folding affect both motor protein function and MT dynamics causing defects in axonal motility.

Our observations also propose a mechanism in which α-syn-mediated axonal transport defects instigate downstream phenotypes such as synaptic abnormalities and locomotion impairments. Previous work showed that α-syn expression decreased the levels of synaptic proteins together with synaptic vesicle densities (Nemani et al., 2010). We found that excess α-syn not only decreased the localization of synaptic proteins CSP and BRP to the synapse, but also influenced the larval NMJs morphology, by increasing the number of satellite boutons (Figure 2 and Supplementary Figures S4, S5). α-Syn overexpression also affected neurite growth and branching in rat primary midbrain neurons (Koch et al., 2015), B103 cells (Takenouchi et al., 2001), and primary hippocampal neurons (Sousa et al., 2009). Viral expression of α-syn in the substantia nigra (SN) of mice resulted in a decrease of rabphilin 3A and syntaxin, other proteins involved in synaptic transmission and kinesin (Chung et al., 2009). Since CSP and BRP are suggested to be required for synaptic transmission (Arnold et al., 2004; Wagh et al., 2006) and synaptic function (Kang et al., 2014), the α-syn-mediated axonal transport defects we observed could directly contribute to the synaptic morphology and larval locomotion defects seen in α-syn-expressing larvae. Indeed, the larval locomotion defects we observe in larvae expressing excess α-syn were similar to locomotion defects seen in larvae with 50% reduction of motor proteins (Figure 3 and Supplementary Figure S6). Correlation analysis of larval locomotion and α-syn-mediated axonal accumulations demonstrate that these larval crawling defects are likely related to the extent of axonal accumulations (Figure 3F). Similarly, motor behaviors in transgenic A53T mice correlated with the level of dynein in their striatum (Liu et al., 2014). Therefore, axonal transport defects likely precede synaptic morphological defects and the behavioral deficits associated with PD (Chung et al., 2009; Chu et al., 2012), consistent with what was proposed for two other neurodegenerative diseases (Kang et al., 2014).

The central hydrophobic region of α-syn, the NAC region, spanning residues 61-95 is known to be essential for its aggregation (Giasson et al., 2001; Bodles et al., 2001; Periquet et al., 2007). Solid-state and solution NMR spectroscopy showed that this region can modulate the affinity of α-syn association with cellular membranes (Fusco et al., 2014). Although α-syn lacks a transmembrane domain and other membrane anchors, studies showed that residues 61-95 can adopt a helical conformation that can partially embed in micelle, lipid molecules that arrange themselves in spherical forms in aqueous solutions (Bisaglia et al., 2005, 2006), suggesting that the structural conformation of this region could instigate interactions with membranes. Interestingly, the NAC region has been proposed to be required for α-syn membrane translocation (Lee et al., 2005), and we found that deletion of the NAC region not only decreased its transport to larval NMJs, with increased levels in the larval brain (Figure 4), and prevented the α-syn-mediated synaptic morphological and larval locomotion defects (Figure 5 and Supplementary Figures S8–S10), but also decreased the amount of α-syn in the membrane fraction (Figure 6). Therefore, the NAC region of α-syn is essential to facilitate associations with membranes under physiological conditions.

Several *in vitro* studies suggest that dimerization of α-syn is critical for α-syn binding to vesicles and for α-syn self-assembly (Colebc et al., 2002; Jo et al., 2002; Giannakis et al., 2008; Roostaee et al., 2013). However, whether this is indeed the case *in vivo* is unclear. Hydrophobic interactions between two NAC regions promoted dimer formation, indicating that dimeric α-syn species can initiate α-syn self-assembly leading to higher order oligomers (Lv et al., 2015, 2016). Intriguingly, fPD mutants A53T, E46K, or A30P enhance the lifespan of dimers and stabilizes them (Lv et al., 2015), causing an increase in the propensity for α-syn aggregation *in vitro* (Breydo et al., 2012). Perhaps the increased amounts of α-syn axonal accumulations we observed *in vivo* (Figure 1) and the suppression of such accumulations and rescue of α-syn-mediated synaptic defects by the co-expression of delNAC (Figures 7, 8) could likely be due to the titration of α-syn dimerization. Indeed, consistent with this proposal expression of delNAC with α-syn^WT^ or excess α-syn significantly decreased the ratio of HMW/LMW species of α-syn (Supplementary Figure S13) indicating that the NAC region likely plays an important role. However, whether such physiological conformations of α-syn are also required for proper membrane binding to vesicles *in vivo* is unclear. Further, our *in vivo* observations are consistent with other previous work that showed that expression of α-syn, a non-amylogenic homolog of α-syn lacking the NAC region, inhibits neuronal α-syn accumulation in mice (Hashimoto et al., 2001). Intracerebral lentiviral injection studies of α-syn in transgenic α-syn mice also decreased α-syn inclusions (Hashimoto et al., 2004). Therefore, under physiological conditions, dimerization of α-syn mediated by the NAC region likely plays an important role in the binding of α-syn to membranes and in the proper transport of axonal α-syn *in vivo*. While future studies are needed to test predictions of this proposal, our work demonstrates the importance of the NAC region during axonal transport. Our work also highlights a potential novel therapeutic pathway that involves the use of delNAC as an early treatment strategy for PD, prior to neuronal loss and behavioral defects.

## Materials and Methods

### *Drosophila* Genetics

The UAS-α-syn^WT^ and α-syn^Δ71–82^ lines were obtained from Dr. Mel Feany (Periquet et al., 2007). α-Syn^LP3^ and TH-GAL4 lines were obtained from Dr. Leo Pallanck (Trinh et al., 2008). The w^∗^; P {UAS-GFP.S65T} (referred to as UAS-GFP) and P{UAS-HsapSNCA.A53T} (referred to as UAS-α-syn^A53T^) were obtained from Bloomington *Drosophila* Stock center. The w;UAS-α-syn^WT^-EGFP/TM6B line was a kind gift from Dr. Pedro M. Domingos (Poças et al., 2014). Loss of function kinesin, *yw; khc^20^/CyO* and *klc^8ex94^/TM6B*, and dynein, *robl^*k*^/T (2:3) CyO TM6B*, Tb and *Dhc^6–10^/TM6B* lines were previously described (Gunawardena and Goldstein, 2001; Gunawardena et al., 2003). All *Drosophila* lines were reared at room temperature. The pan neuronal APPL-GAL4 (Gunawardena and Goldstein, 2001; Gunawardena et al., 2003) or the dopaminergic driver, TH-GAL4 (Trinh et al., 2008) were used to express human α-syn protein (at 29°C). For the generation of α-syn^Δ71–82^ in the context of α-syn^WT^ or α-syn^*LP2*^, first virgins from the UAS-α-syn^Δ71–82^ line were crossed to males that were APPL-GAL4; *T(2:3)CyO TM6B*/Pin (Gunawardena and Goldstein, 2001; Gunawardena et al., 2003). Males that were APPL-GAL4: UAS-α-syn^Δ71–82^/*T (2:3) CyO* TM6B were then crossed to virgins from UAS-α-syn^WT^ or UAS-α-syn^LP3^ and female larvae that were APPL-GAL4: UAS-α-syn^Δ71–82^/UAS-α-syn^WT^ or UAS-α-syn^LP3^ were dissected or harvested for experiments. Only 25% of the progeny from this cross will be APPL-GAL4: UAS-α-syn^Δ71–82^/UAS-α-syn^WT^ or UAS-α-syn^LP3^ females.

### Larval Preparation, Immunohistochemistry, Imaging, and Quantification

Third instar larvae were dissected, fixed, and segmental nerve immunostainings were done as previously described (Kang et al., 2014). Briefly, larvae were dissected in dissection buffer (2X stock contains 128 mM NaCl, 4 mM MgCl_2_, 2 mM KCl, 5 mM HEPES, and 36 mM sucrose, pH 7.2). Dissected larvae were fixed in 4% formaldehyde and incubated with antibodies against CSP (CSP, 1:10, Developmental Studies Hybridoma Bank), α-syn (1:100, Invitrogen), BRP (1:10, Developmental Studies Hybridoma Bank), kinesin light chain (KLC, 1:50, Goldstein), pSer/Thr (1:100, Cell Signaling Technology), and/or HRP-TR or HRP-FITC (1:100, Invitrogen) overnight. Larvae were incubated in secondary antibodies (Alexa-568-conjugated-anti-mouse or Alexa-488-conjugated-anti-rabbit, 1:100, Invitrogen) and mounted using Vectashield mounting medium (Vector Labs). Images were collected using Leica TCS SP2 AOBS Spectral confocal microscope or Nikon Eclipse TE 2000-U inverted microscope with a Coolsnap HQ camera as described (Gunawardena and Goldstein, 2001; Gunawardena et al., 2003). All quantification was done using a blinded or double-blinded protocol. Quantitative analysis on α-syn or CSP accumulation in larval brains, larval segmental nerves, and larval NMJs between muscle 6/7 and muscle 4 at abdominal segments A2 or A3 was carried out by collecting six to eight confocal optical images. For α-syn or CSP accumulation in larval brain and segmental nerves, six to seven animals were quantified for each genotype, using the NIH image software as previously described (Kang et al., 2014). For NMJ analysis, NMJs between muscles 6/7 and muscle 4 at segments A2 or A3 from 10 to 15 animals were imaged. The threshold, density slice, and particle analysis function in NIH image software were used to quantify parent and satellite boutons, bouton areas, and synaptic length. Average numbers of parent and total boutons, and synaptic lengths and the% of satellite boutons were calculated and graphed using an Excel worksheet. Using NIH image, α-syn, CSP, and BRP intensities were obtained and graphed using an Excel worksheet. The one-way ANOVA and the two-tailed Student’s *t*-tests were performed using Excel (Microsoft Corp.). Differences were considered significant at a significance level of 0.05, which means a 95% statistically significant correlation. Error bars represent standard error of the mean (SEM).

### Larval Locomotion Assays

Ten third instar larvae from each genotype were collected, and stationed in a food environment until the start of the crawling assay. To motivate the larva to crawl, a scoop of fresh food was placed at the finish line. The track on which the larvae crawled was lubricated with buffer. The larva was allowed to crawl a distance of 2 in without distractions and the time was recorded. The crawling velocity was calculated and the average was calculated from 10 independent larval crawling from each genotype. For the “contractile assay,” the number of full body peristaltic contractions in 1 min was recorded. For each genotype, 10 independent larvae were used. Three trials were done for both crawling and contractile assay and the averaged was used. Using an Excel worksheet, larval locomotion from larvae expressing the different α-syn variants or GFP using APPL-GAL-4 or TH-Gal4 and larvae carrying heterozygous and homozygous motor proteins mutations were calculated by normalizing the crawling and contractile assay values to WT. Thus, WT larvae were set to 1. Statistical analysis was carried out using the one-way ANOVA and the two-tailed Student’s *t*-tests. Differences were considered significant at a significance level of 0.05, which means a 95% statistically significant correlation. Error bars represent SEM.

### Western Blot Analysis, Membrane Flotation Assay, and Native Gel Electrophoresis

#### Western Blot

As previously described 50 fly heads from each genotype (α-syn^WT^, α-syn^LP3^, and α-syn^A53T^, α-syn^Δ71–82^ and WT) were homogenized in Buffer A [250 mM sucrose, 15 mM Tris-HCL (pH 7.9), 60 mM KCL, 15 mM NaCL 1 mM EGTA, 5 mM EDTA, 0.5 mM DTT] with proteinase and phosphotase inhibitors (Gunawardena et al., 2003) for Western blot. Debris were removed by centrifugation at 1,000×*g* for 7 min. Antibodies α-syn monoclonal (LB509, Invitrogen at 1:1,000) and anti-tubulin monoclonal antibody (Abcam at 1:1,000 dilution) were used. Immunoreaction was detected using the ECL kit (Pharmacia) and imaged using Quantity One (Bio-Rad). Quantification analysis was performed using NIH ImageJ software. Data obtained as percent values for each sample by Image J were analyzed in Excel (Microsoft Corp.). Relative intensities were calculated by dividing the percent value for each sample by percent value of tubulin and then normalized to α-syn^WT^. Four separate membranes from four independent experiments were used for quantification.

#### Native-Non-denaturing Gel Electrophoresis

To detect aggregation, more than 125 (>25 for α-syn^WT^;α-syn^Δ71–82^ and α-syn^LP2^;α-syn^Δ71–82^) fly heads per genotype (α-syn^WT^, α-syn^LP3^, α-syn^A53T^, α-syn^Δ71–82^, and WT) at 20 days old were homogenized in Buffer A [250 mM sucrose, 15 mM Tris-HCL (pH 7.9), 60 mM KCL, 15 mM NaCL 1 mM EGTA, 5 mM EDTA, 0.5 mM DTT] with proteinase and phosphotase inhibitors (Gunawardena et al., 2003). The homogenate was centrifuged at 16,000 *g* for 15 min (Periquet et al., 2007) and the resulting supernatant was analyzed by Native-PAGE 10% Tris-glycine gel and immunoblot with α-syn monoclonal (LB509, Invitrogen at 1:1,000) and anti-tubulin monoclonal antibody (Abcam at 1:1,000 dilution) were used. Immunoreaction was detected using the ECL kit (Pharmacia) and imaged using Quantity One (Bio-Rad). Quantification analysis of HMW (>250 kDa) and LMW (between 80 and 60 kDa) was performed using Image Lab (Bio-Rad) software on two independent experiments. Data obtained from Image Lab were analyzed and plotted using Origin 2019b software. Relative ratio of intensity was calculated by dividing the HMW α-syn intensity value by the LMW α-syn intensity value for each genotype and then normalized to α-syn^WT^.

#### Membrane Floatation Assay

As previously described, larval brains from each genotype (WT, α-syn^WT^, α-syn^LP3^, α-syn^Δ71–82^) were homogenized in acetate buffer (10 mM HEPES, pH 7.4, 100 mM K acetate, 150 mM sucrose, 5 mM EGTA, 3 mM Mg acetate, 1 mM DTT) with proteinase and phosphatase inhibitors (Dolma et al., 2014). Debris were removed by centrifugation at 1,000×*g* for 7 min and the resulting PNS was brought to 40% sucrose and overlaid with a 35% sucrose and 8% sucrose. The gradient was then centrifuged at 50,000 r/min in a TLS55 swing bucket rotor (Beckman Coulter, Fullerton, CA, United States) for 1 h. Light membranous organelles and membrane associated proteins floated at the 35/8 fraction. Equal amounts of protein from the PNS, 35/8 fraction, and soluble fractions were analyzed by Western blotting. Immunoreaction was detected using the ECL kit (Pharmacia) and imaged using Quantity One (Bio-Rad). Quantification analysis was performed using NIH ImageJ software. Data obtained as percent values for each sample by Image J were analyzed in Excel (Microsoft Corp.). Relative intensity was calculated by dividing the percent value for each sample by percent value of Rab5 and then normalized to α-syn^WT^. Using the two-sample two-sided Student’s *t*-test (and the Bonferroni’s test and Tukey’s HSD tests), differences were considered significant at a significance level of 0.05, which means a 95% statistically significant correlation from four separate membranes from four independent experiments.

#### Co-immunoprecipitation Assay

Half a WT mouse brain was homogenized in acetate buffer as previously described (Banerjee et al., 2018). The lysate was centrifuged at 1,000 *g* for 10 min at 4°C. Concentrations of the extracts were determined using BCA (Bicinchoninic acid) protein assay (Pierce). For IP, 2 mg of the lysate was incubated overnight with 4 μg α-syn antibody (BD Biosciences) at 4°C. Protein A/G Magnetic Beads (Pierce) washed in wash buffer (Tris-buffered saline containing 0.05% Tween-20) was added to the mixture and incubated at room temperature for 1 h. Magnetic beads were then eluted in 100 μL low pH elution buffer (Pierce). The low pH was neutralized by adding 15 μL Tris pH 8.8. The concentration of the α-syn pull down was determined by BCA assay. α-Syn immunoprecipitation was performed using α-syn monoclonal antibody (BD Biosciences at 1:1,000). Western blot analysis was used to evaluate the extent and purity of the α-syn immunoprecipitation as well as whether kinesin-1 (KIF5C polyclonal antibody at 1:250) and dynein (DIC, Abcam at 1:200) pulled down with the α-syn immunoprecipitation.

### Statistical Analysis

Statistical analysis was preformed using GraphPad Prism 6 software and Minitab 18. First power and sample size calculations were performed: comparing two means from two samples, with two-sided equality to identify the sample size that corresponds to a power of 0.95 with α = 0.05. Calculation of sample size for a power of 0.95 and α = 0.05 indicated a sample size of five larvae was necessary. For immunofluorescence analysis of axonal blockages, statistical analysis was performed using the two-sample two-sided Student’s *t*-test and one-way ANOVA test. Differences were considered significant at a significance level of 0.05, which means a 95% statistically significant correlation for 5–10 individual larvae from several independent crosses. For western blots, quantification analysis was performed using Image Lab software. Data obtained from Image Lab were analyzed in Excel (Microsoft Corp.) using two-sided Student’s *t*-test. Additionally, Bonferroni’s and Tukey’s HSD tests were also performed in Minitab 18. Both of these methods are pair-wise multiple comparison procedures specifically designed to compare each treatment with a control. Differences were considered significant at a significance level of 0.05, which means a 95% statistically significant correlation from three separate membranes from three independent experiments.

## Data Availability Statement

All datasets generated for this study are included in the article/Supplementary Material.

## Author Contributions

EA and SG conceived the project, designed the research, analyzed data, and wrote the manuscript. EA, DH, KZ, and RB performed the experiments and analyzed the data.

## Conflict of Interest

The authors declare that the research was conducted in the absence of any commercial or financial relationships that could be construed as a potential conflict of interest.
