# Supplementary material for: The Non-amyloidal Component Region of α-Synuclein Is Important for α-Synuclein Transport Within Axons
Source: Front Cell Neurosci. 2020 Jan 10;13:540. doi: 10.3389/fncel.2019.00540 (PMC6984405; doi:10.3389/fncel.2019.00540)
Supplement: Supplementary file 1 [file Presentation_1.pdf]

Figure S1:

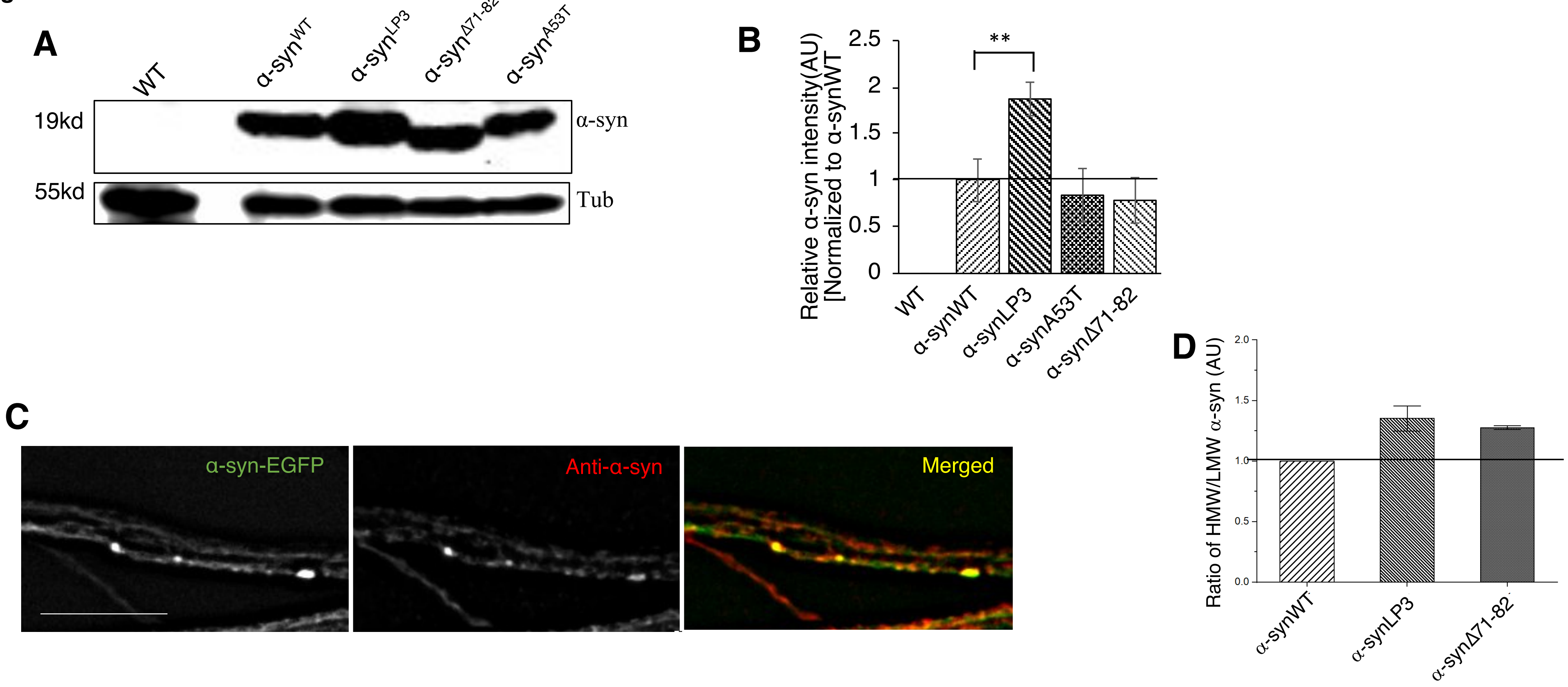

Figure S1: Expression of α-syn in *Drosophila* (**A**) Western blot analysis of α-syn protein levels in flies expressing α-syn^{WT}, α-syn^{LP3}, α-syn^{A53T}, and α-syn lacking regions 71-82 (α-$syn^{\Delta71\text{-}82}$). Tubulin was used as a loading control. **(B)** Quantification of the relative intensity of α-syn (AU= arbitrary units) indicates a significantly higher level of α-syn protein in α-syn^{LP3} compared to α-syn^{WT} ($p<0.01$), while α-syn^{A53T} or α-$syn^{\Delta71\text{-}82}$ showed no significant differences in the amount of α-syn protein. Quantitative analysis represents mean ± SEM. **$p<0.01$, n = 3, Bar = 25 µm. **(C)** Image of EGFP tagged wild type α-syn (α-syn^{WT}-EGFP, green) stained with α-syn antibody (red) show co-localization (yellow) in larval segmental nerve. (**D**) Quantification of the ratio of high molecular weight (HMW) insoluble and low molecular weight (LMW) soluble α-syn species from native non-denaturing gel electrophoresis of α-syn^{WT}, α-syn^{LP3} and α-syn^{A53T} normalized to α-syn^{WT}. Note that α-syn^{LP3} or α-syn^{A53T} show increased ratio of HMW/LMW α-syn compared to α-syn^{WT}. n=2 independent membranes.

Figure S2:

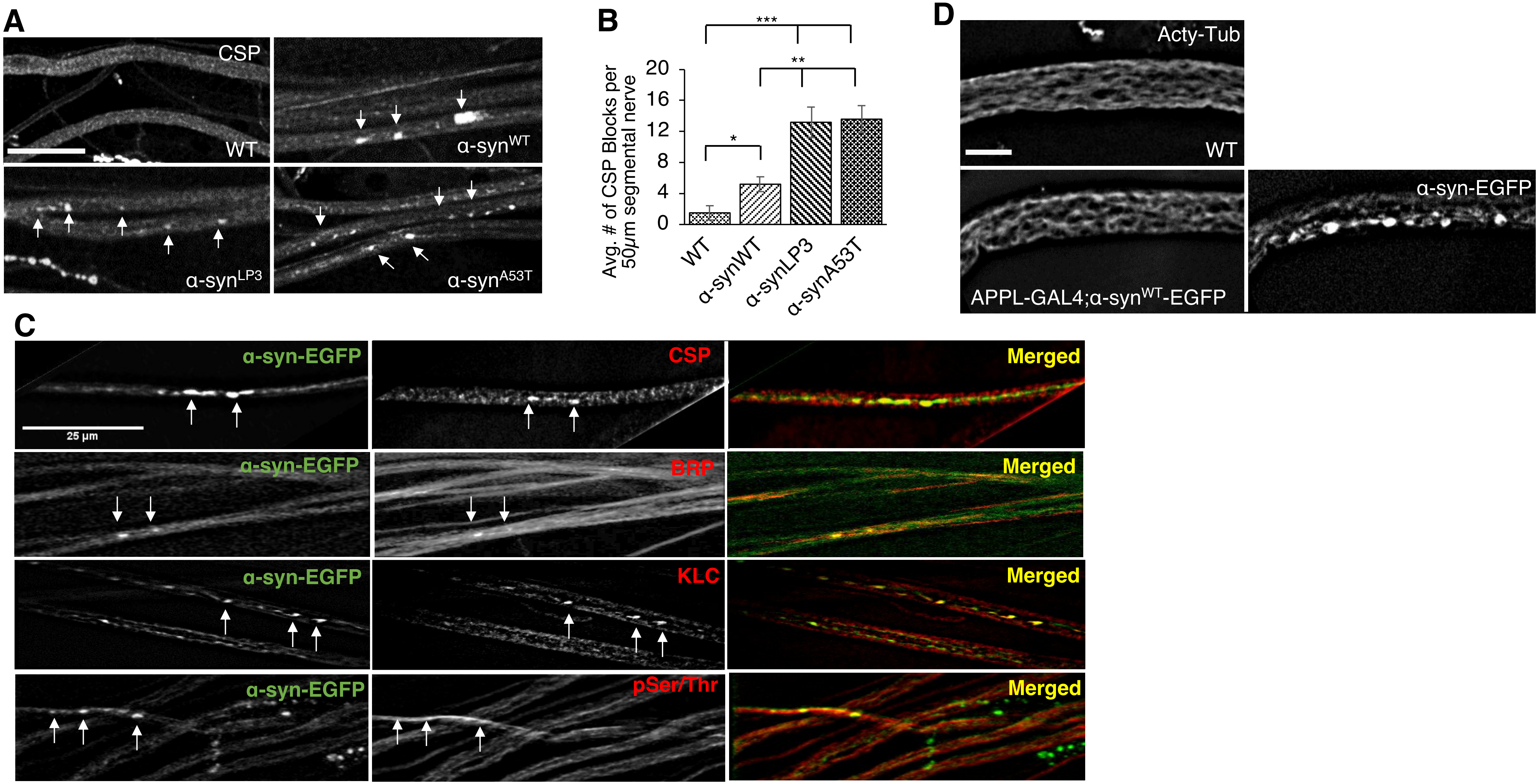

A
CSP
WT
α-syn^{WT}
α-syn^{LP3}
α-syn^{A53T}
B
***
**
*
Avg. # of CSP Blocks per 50μm segmental nerve
20
16
12
8
4
0
WT
α-synWT
α-synLP3
α-synA53T
C
α-syn-EGFP
CSP
Merged
25 μm
α-syn-EGFP
BRP
Merged
α-syn-EGFP
KLC
Merged
α-syn-EGFP
pSer/Thr
Merged
D
Acty-Tub
WT
APPL-GAL4;α-syn^{WT}-EGFP
α-syn-EGFP

Figure S2: Expression of α-syn causes axonal transport defects and perturbs the motility of synaptic proteins CSP and BRP. (A) Larval segmental nerves expressing α-syn^{WT}, α-syn^{LP3}, or α-syn^{A53T} show axonal blockages (arrow) as assayed by the synaptic vesicle protein, cysteine string protein (CSP), while WT nerves are smoothly stained. **(B)** Quantitative analysis indicates that α-syn^{WT} ($p<0.05$), α-syn^{LP3} ($p<0.001$), or α-syn^{A53T} ($p<0.001$) larval segmental nerves contain a significantly higher number of axonal blocks compared to WT. α-syn^{LP3} and α-syn^{A53T} also show a significant increase in axonal blocks compare to α-syn^{WT} ($p<0.01$). (**C)** α-syn-EGFP (Green) and CSP (red), BRP and KLC co-localize on axonal blocks (arrows) within larval segmental nerves. Note that α-syn-EGFP-containing axonal blocks are also positive for pSer/Thr antibody (red) arrows. **(D)** Larvae expressing α-syn^{WT}-EGFP stained with acetylated tubulin show that the microtubules are intact and are similar to WT controls. Quantitative analysis represents mean ± SEM. *$p<0.05$, **$p<0.01$, ***$p<0.001$, n =7, Bar = 25 μm.

Figure S3:

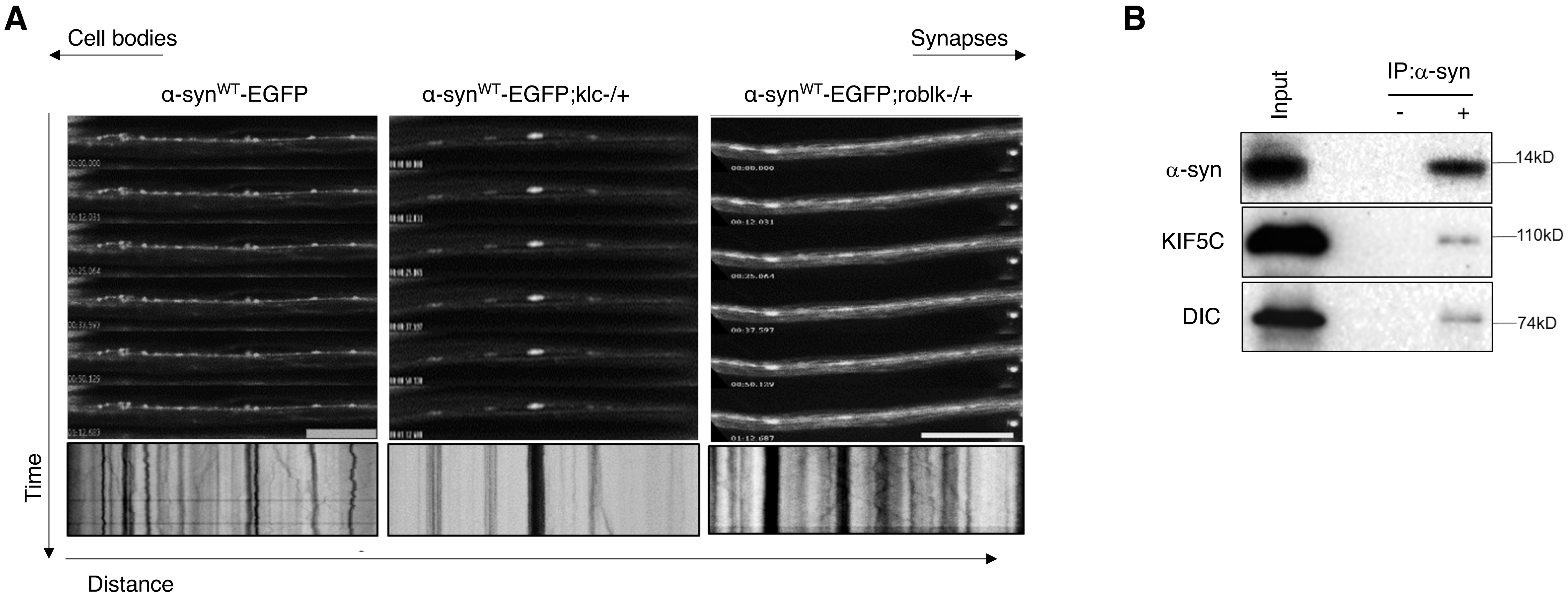

Figure S3: The bi-directional movement of α-syn requires kinesin-1 and dynein molecular motors. (A) A representative movie montage from larvae expressing α-syn-EGFP and the corresponding kymograph depicts anterograde and retrograde motility of α-syn. The direction of cell bodies and synapses are depicted with arrows. The Y-axis depicts time in seconds while the X-axis depicts distance travelled in microns. Note that 50% reduction of kinesin or dynein with α-syn-EGFP shows α-syn-EGFP-containing accumulations which are stalled in the kymograph. Bar=10µm. (B) Co-IP analysis of α-syn and molecular motors from mouse brains. α-syn was first IPed using an α-syn antibody. Input shows the extent of α-syn protein. Antibodies against kinesin-1 (KIF5C) and dynein (DIC) are present with α-syn. Negative (-) lane is the no antibody control.

Figure S4:

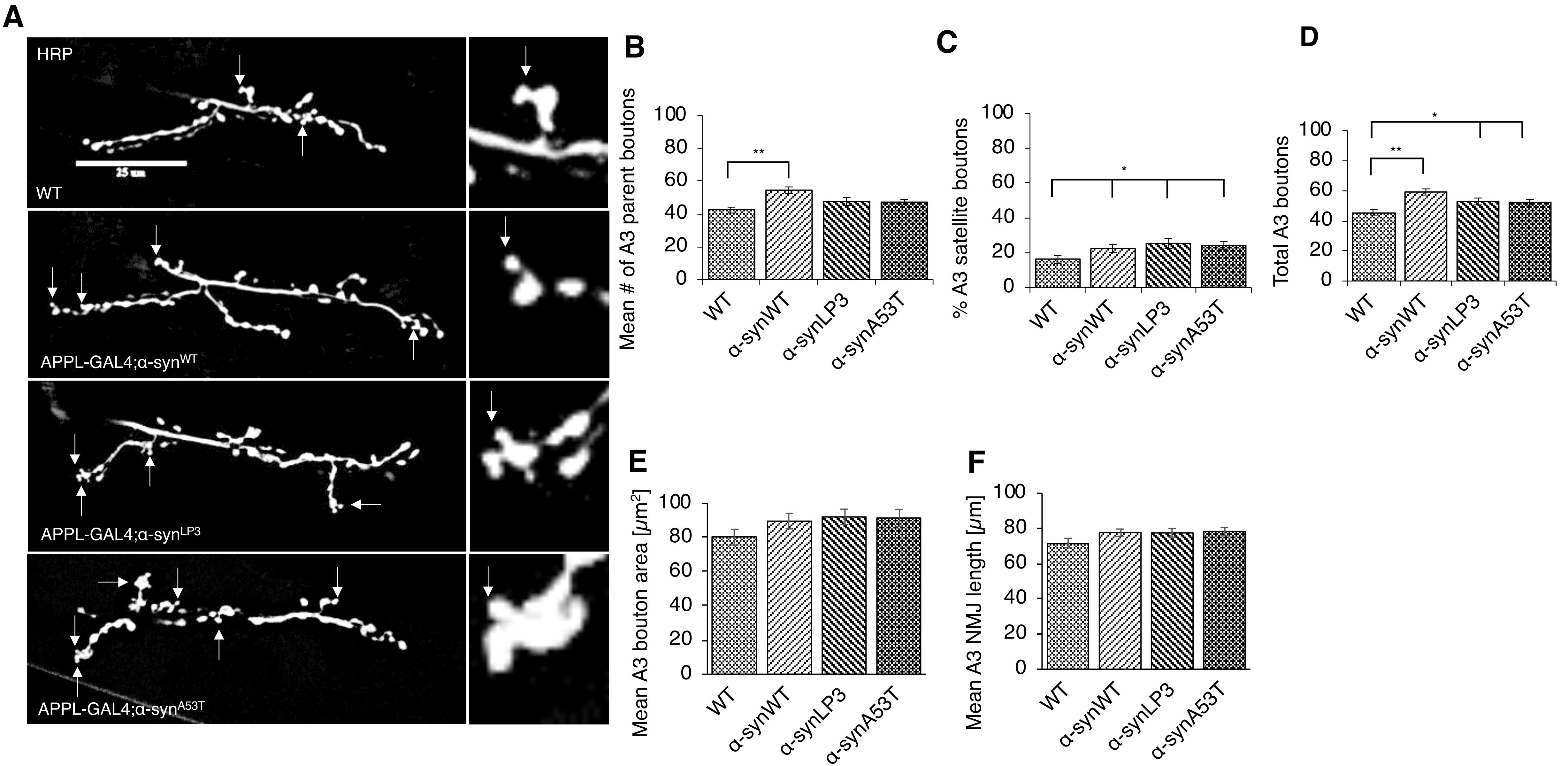
A
HRP
25 um
WT
APPL-GAL4;α-syn^WT
APPL-GAL4;α-syn^LP3
APPL-GAL4;α-syn^A53T
B
Mean # of A3 parent boutons
100
80
60
40
20
0
**
WT
α-synWT
α-synLP3
α-synA53T
C
% A3 satellite boutons
100
80
60
40
20
0
*
WT
α-synWT
α-synLP3
α-synA53T
D
Total A3 boutons
100
80
60
40
20
0
*
**
WT
α-synWT
α-synLP3
α-synA53T
E
Mean A3 bouton area [μm²]
100
80
60
40
20
0
WT
α-synWT
α-synLP3
α-synA53T
F
Mean A3 NMJ length [μm]
100
80
60
40
20
0
WT
α-synWT
α-synLP3
α-synA53T

Figure S4: Expression of α-syn causes synaptic morphological defects. (**A**) Larval NMJ from muscle 6/7 from segment A3 from control and larvae expressing α-syn stained with the pre-synaptic marker, HRP. Insert shows a representative parent bouton with satellite boutons (arrows). (**B**) Quantification of the mean number of parent boutons indicate a significant increase in α-syn^{WT} larvae compared to WT ($p<0.01$), while no changes were seen in α-syn^{LP3} or α-syn^{A53T} larvae. (**C**) Quantitative analysis of the percent (%) satellite boutons show significant increases in α-syn^{WT}, α-syn^{LP3}, and α-syn^{A53T} larvae as compared to WT ($p<0.05$). **(D)** The total number of boutons was significantly increased in α-syn^{WT} ($p<0.01$), α-syn^{LP3} ($p<0.05$) and α-syn^{A53T} ($p<0.05$) compare to WT. Note that the total number boutons include both parent and satellite boutons. Quantification analysis of bouton area (**E**) or NMJ length (**F**) in α-syn^{WT}, α-syn^{LP3}, or α-syn^{A53T} larvae show no differences compared to WT. Quantitative analysis represents mean ± SEM. *$p<0.05$, **$p<0.01$, **$p<0.001$, n= 10, Bar = 25µm

Figure S5:

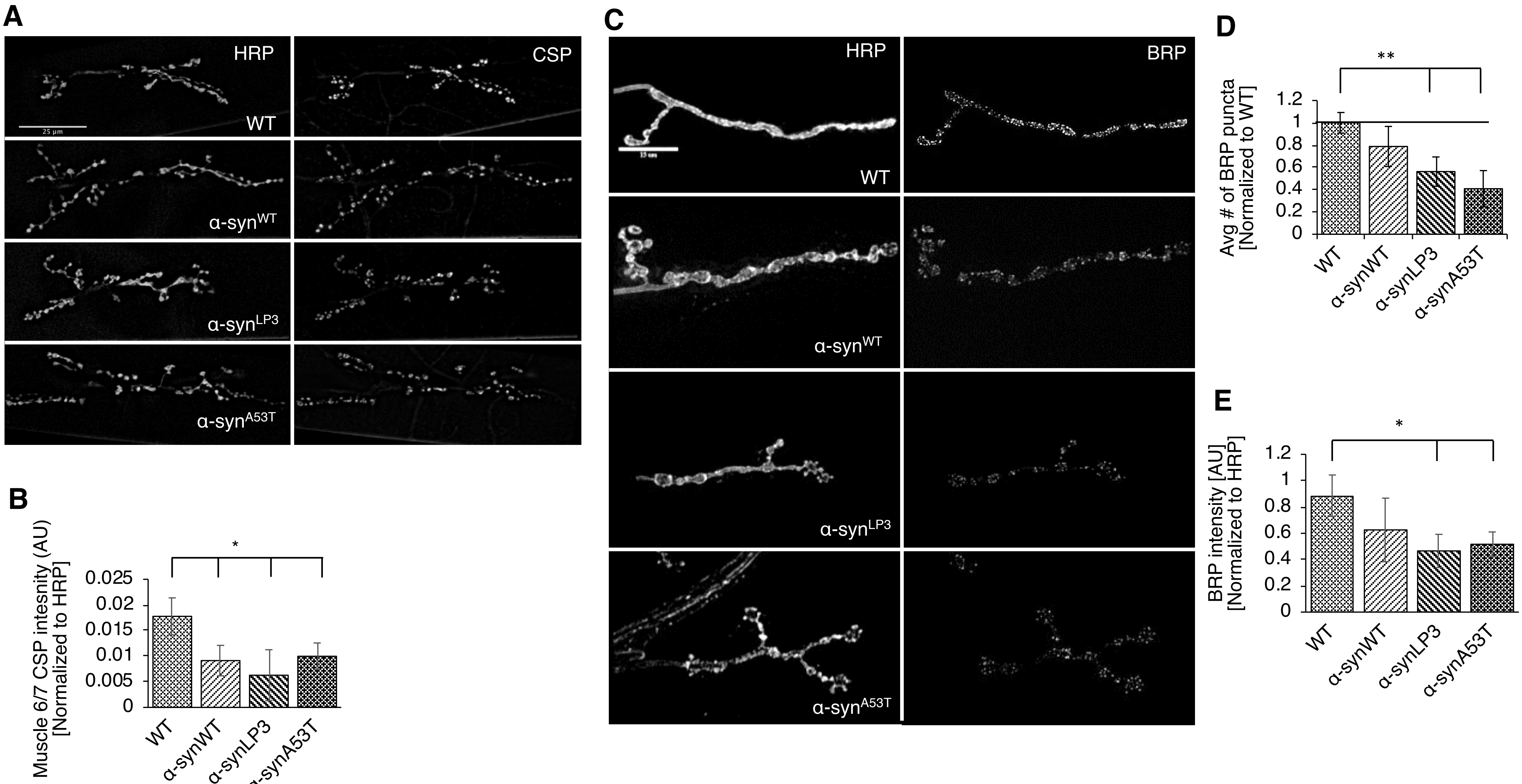
A
HRP
CSP
WT
25 µm
α-syn^{WT}
α-syn^{LP3}
α-syn^{A53T}
B
Muscle 6/7 CSP intesnity (AU) [Normalized to HRP]
*
0.025
0.02
0.015
0.01
0.005
0
WT
α-synWT
α-synLP3
α-synA53T
C
HRP
BRP
WT
α-syn^{WT}
α-syn^{LP3}
α-syn^{A53T}
D
**
Avg # of BRP puncta [Normalized to WT]
1.2
1
0.8
0.6
0.4
0.2
0
WT
α-synWT
α-synLP3
α-synA53T
E
*
BRP intensity [AU] [Normalized to HRP]
1.2
1
0.8
0.6
0.4
0.2
0
WT
α-synWT
α-synLP3
α-synA53T

Figure S5: Expression of α-syn reduced synaptic proteins levels at the NMJ. (**A**) Larval NMJs from muscle 6/7, segment A2 from larvae expressing α-syn or WT stained with HRP and CSP show decreases in synaptic staining (Bar = 25μm). (**B**) Quantitative analysis of CSP intensity (normalized to HRP) show significant decreases in CSP intensities in α-syn^{WT}, α-syn^{LP3}, and α-syn^{A53T} expressing larval NMJs compare with WT ($p<0.05$). (**C**) Larval NMJs from segment A2 of muscle 4 stained with HRP and active zone marker Bruchpilot (BRP) in α-syn^{WT}, α-syn^{LP3}, α-syn^{A53T}, and WT larvae also show decreases in active zone staining. (**D**) Quantification of the normalized average BRP puncta show a significant reduction in α-syn^{LP3} ($p<0.01$) and α-syn^{A53T} ($p<0.01$), but not in α-syn^{WT} larval NMJs compare to WT (Bar = 15μm). (**C**) Quantification of the fluorescence intensity of BRP also show significant decreases in α-syn^{LP3} ($p<0.01$) and α-syn^{A53T} ($p<0.01$), but not α-syn^{WT} larval NMJs compared to WT. Quantitative analysis is mean ± SEM. *$p<0.05$, **$p<0.01$, $n= 12$.

Figure S6:

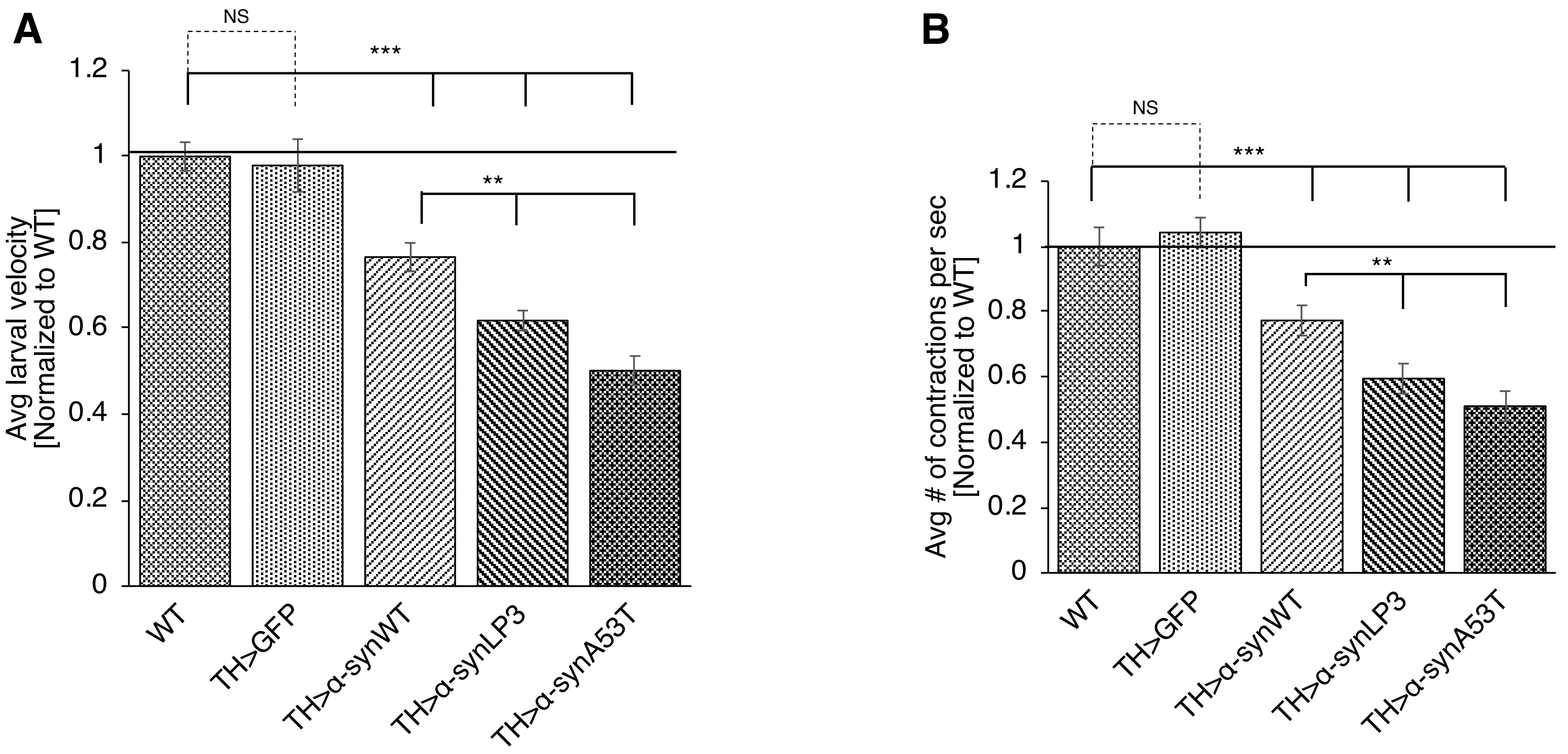

Figure S6: Expression of α-syn in *Drosophila* dopaminergic neurons perturb larval locomotion. (**A**) Quantification of the average crawling velocity from larvae expressing α-syn or GFP normalized to WT using the tyrosine hydroxylase driver which expresses specifically in the dopaminergic neurons in *Drosophila*, TH-GAL4. α-syn^{WT}, α-syn^{LP3}, and α-syn^{A53T} expressing larvae show significant decreases in larval crawling velocity compared to WT ($p<0.001$), while larvae expressing GFP alone show no differences. α-syn^{LP3} and α-syn^{A53T} larvae also show significant decreases in larval crawling velocity compared to α-syn^{WT} larvae ($p<0.01$). (**B**) Quantitative analysis of the average number of larval peristaltic contractions in 60 seconds for WT and larvae expressing human α-syn or GFP normalized to WT. Note that the average number of contractions in α-syn^{WT}, α-syn^{LP3} and α-syn^{A53T} expressing larvae are significantly reduced compared WT ($p<0.001$), while larvae expressing GFP alone show no difference. Quantification of larval peristaltic contractions show a significant decrease in α-syn^{LP3} and α-syn^{A53T} expressing larvae compared to α-syn^{WT} expressing larvae ($p<0.01$). Quantitative analysis is mean ± SEM. **$p<0.01$, ***$p<0.001$, n= 10.

Figure S7:

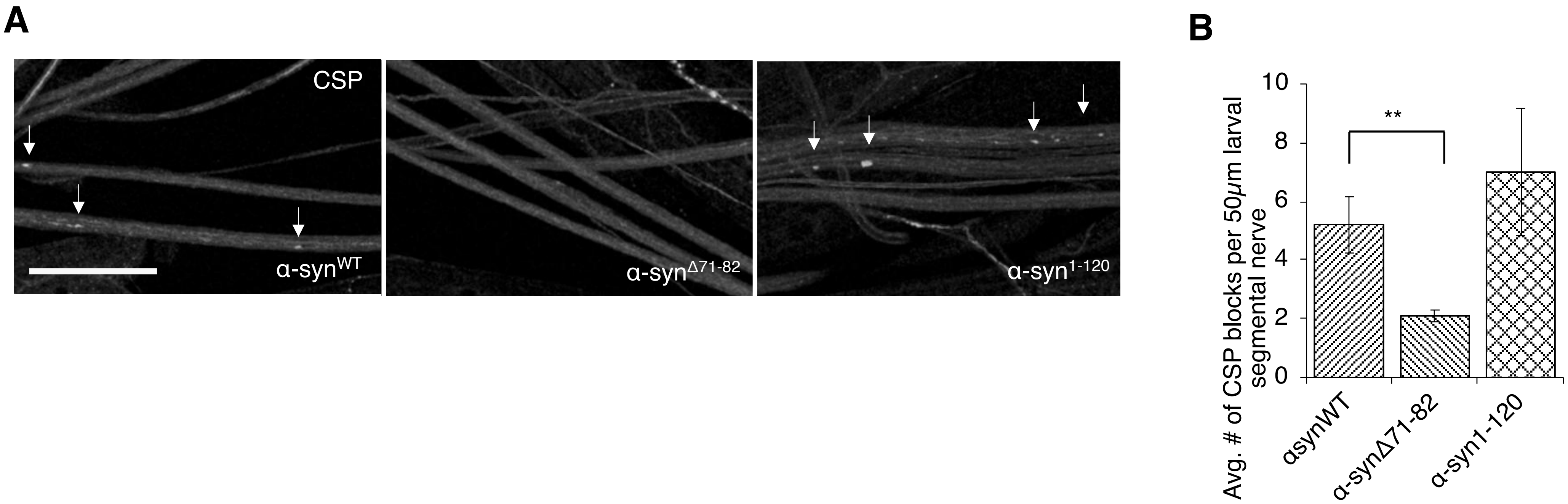

Figure S7: Deletion of the NAC region prevents CSP-positive axonal blockages. (**A**) Segmental nerves from WT, α-syn^{WT}, α-syn$^{\Delta 71\text{-}82}$, and α-syn$^{1\text{-}120}$ expressing larvae stained with CSP. Note that α-syn^{WT} or α-syn$^{1\text{-}120}$ larval segmental nerves contained axonal blocks (arrow) while α-syn$^{1\text{-}120}$ expressing larval nerves are predominantly smoothly stained with CSP, similar to WT. **(B)** Quantitative analysis of CSP blocks indicate that segmental nerves from α-syn$^{\Delta 71\text{-}82}$ larvae contain significantly less axonal blocks compared to α-syn^{WT} ($p<0.01$), while α-syn$^{1\text{-}120}$ show no difference compared with α-syn^{WT}. Quantitative analysis represents the mean ± SEM. **$p<0.01$, (n= 7), Bar = 25μm.

Figure S8:

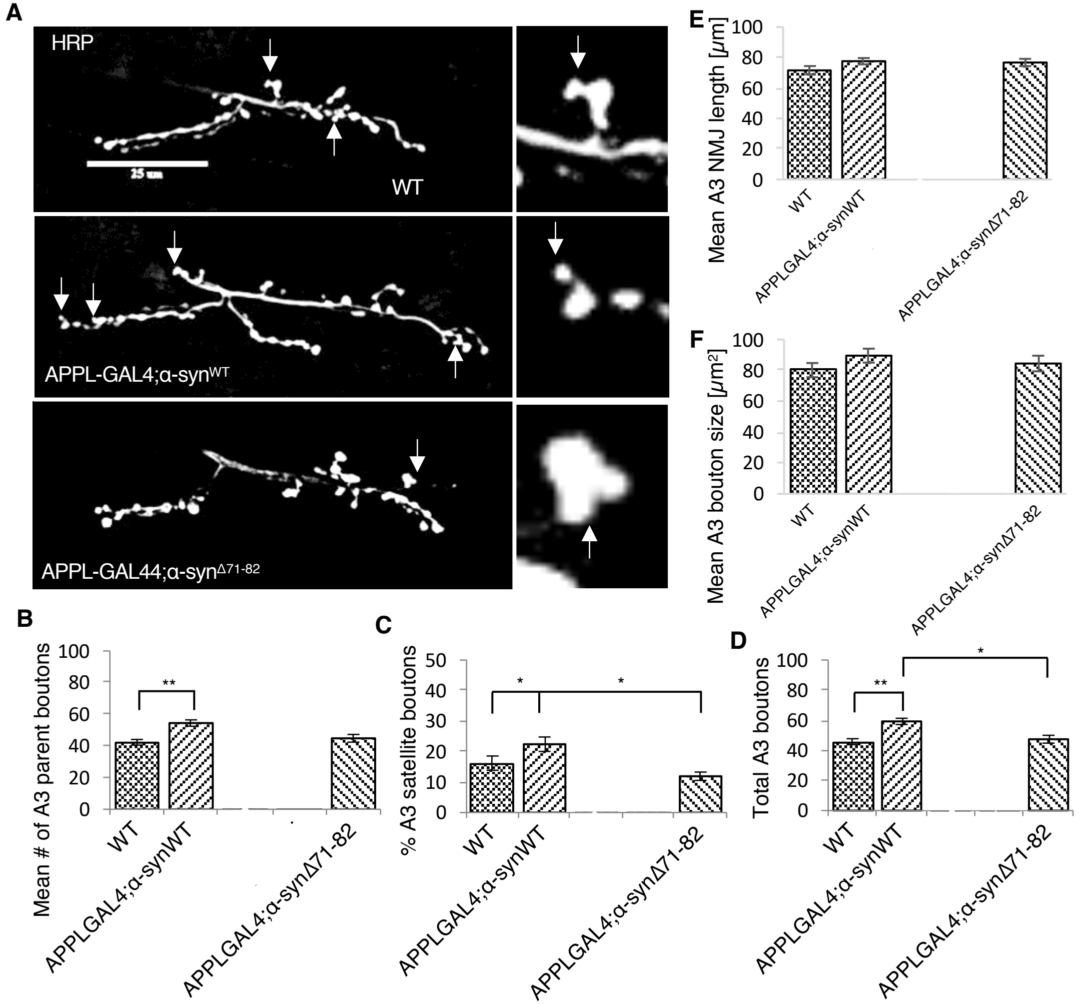

Figure S8: Deletion of the NAC region prevents synaptic morphological defects. (**A**) Larval NMJ from muscle 6/7 from segment A3 from WT and larvae expressing α-syn^{WT} or α-syn$^{\Delta 71-82}$ stained with the pre-synaptic marker, HRP. Insert shows a representative parent bouton with satellite boutons (arrows). (**B**) Quantification of the mean number of parent boutons indicate a significant increase in α-syn^{WT} larvae compared to WT ($p<0.01$), while no changes were seen in α-syn$^{\Delta 71-82}$ larvae which were comparable to WT. (**C**) Quantitative analysis of the percent (%) satellite boutons show significant in α-syn^{WT} larvae compared to WT ($p<0.05$). Note that a significant decrease was seen in α-syn$^{\Delta 71-82}$ compared to α-syn^{WT}. **(D)** The total number of boutons was significantly increased in α-syn^{WT} ($p<0.01$) compare to WT. The total number boutons include both parent and satellite boutons. A significant decrease in the total number of boutons were seen in α-syn$^{\Delta 71-82}$ compared to α-syn^{WT}. Quantification analysis of bouton area (**E**) or NMJ length (**F**) in α-syn^{WT}, or α-syn$^{\Delta 71-82}$ larvae show no differences compared to WT. Quantitative analysis represents mean ± SEM. *$p<0.05$, **$p<0.01$, n= 10, Bar = 25μm

Figure S9:

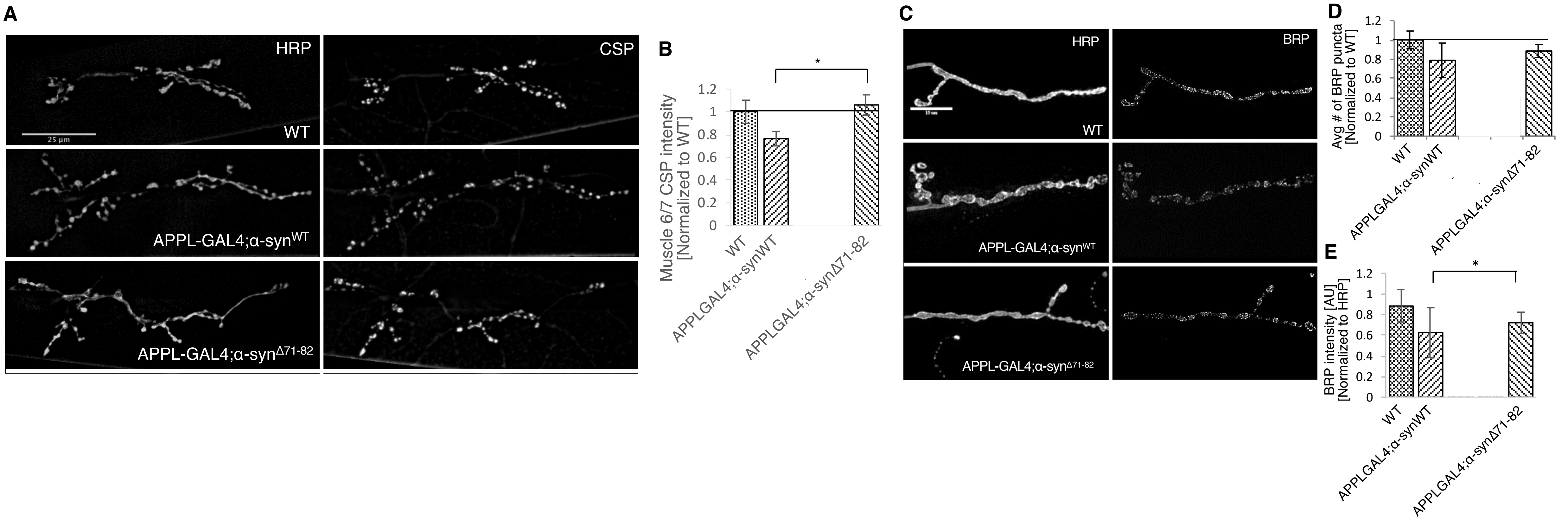

Figure S9: Deletion of the NAC region does not decrease the level of CSP or BRP at the NMJs. (A) Larval NMJs from muscle 6/7, segment A2 from WT larvae and larvae expressing α-syn^{WT} or α-syn$^{\Delta 71\text{-}82}$ stained with HRP and CSP show that α-syn$^{\Delta 71\text{-}82}$ is comparable to WT (Bar = 25µm). (**B**) Quantitative analysis of CSP intensity (normalized to HRP) show significant increases in CSP intensities in α-syn$^{\Delta 71\text{-}82}$ compared to α-syn^{WT} larval NMJs ($p<0.05$). (**C**) Larval NMJs from segment A2 of muscle 4 stained with HRP and active zone marker Bruchpilot (BRP) in WT and α-syn^{WT} or α-syn$^{\Delta 71\text{-}82}$ larvae also show that α-syn$^{\Delta 71\text{-}82}$ is comparable to WT. (**D-E**) While quantification of the normalized average BRP puncta did not show any significant changes, quantification of the fluorescence intensity of BRP showed a significant increase in α-syn$^{\Delta 71\text{-}82}$ larval NMJs compared to α-syn^{WT} larval NMJs ($p<0.05$). Quantitative analysis is mean ± SEM. n= 12.

Figure S10:

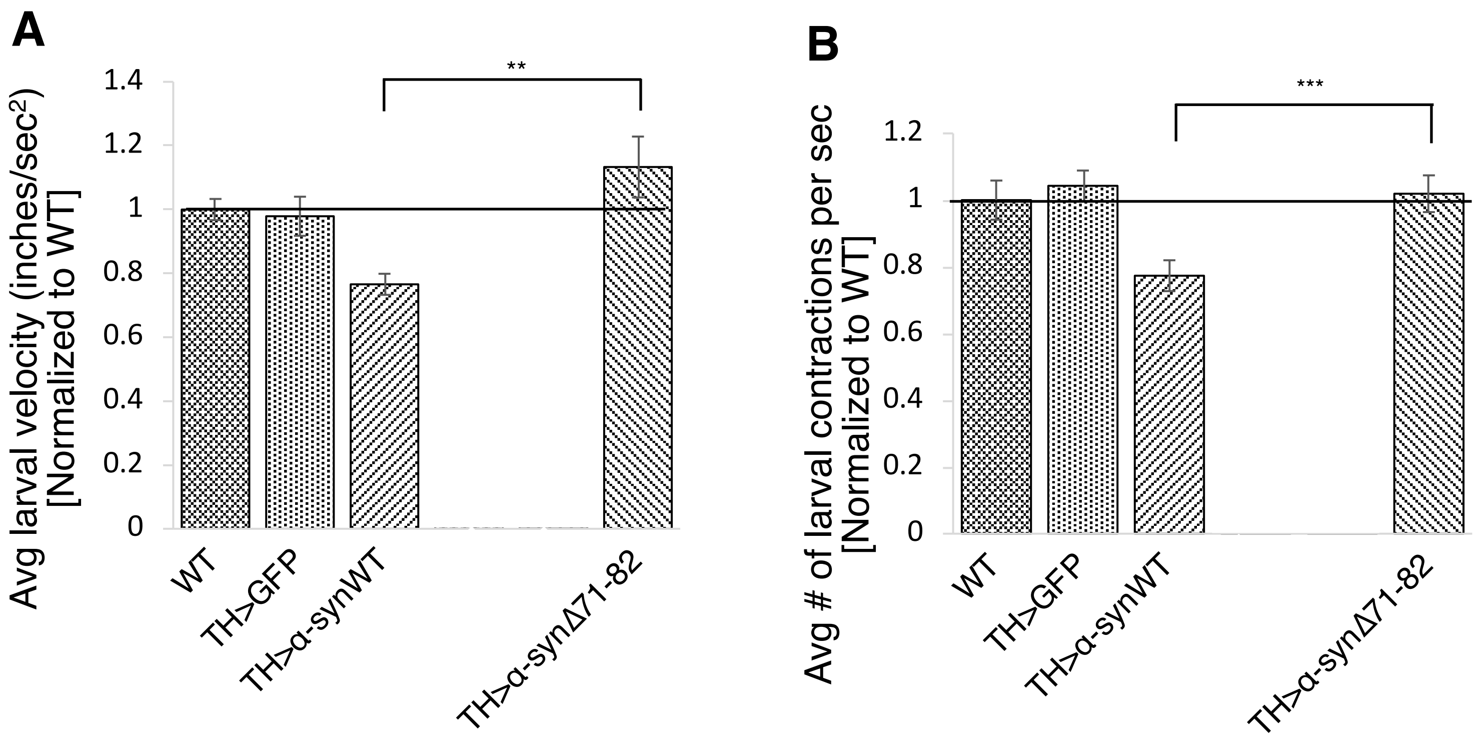

Figure S10: Deletion of the NAC region in *Drosophila* dopaminergic neurons has no effect on larval locomotion. (**A**) Quantification of the average crawling velocity from larvae expressing α-syn^{WT} or α-syn$^{\Delta 71\text{-}82}$ normalized to WT using the tyrosine hydroxylase driver which expresses specifically in the dopaminergic neurons in *Drosophila*, TH-GAL4. α-syn$^{\Delta 71\text{-}82}$ expressing larvae show larval crawling velocities that are compared to WT and are significantly increased compared to α-syn^{WT} ($p<0.01$). (**B**) Quantitative analysis of the average number of larval peristaltic contractions in 60 seconds for larvae expressing α-syn^{WT} or α-syn$^{\Delta 71\text{-}82}$ normalized to WT. Note that the average number of contractions in α-syn$^{\Delta 71\text{-}82}$ are significantly increased compared α- α-syn$^{\Delta 71\text{-}82}$ ($p<0.001$) and are similar to WT. Quantitative analysis is mean ± SEM. **p<0.01, ***p<0.001, n= 10.

Figure S11:

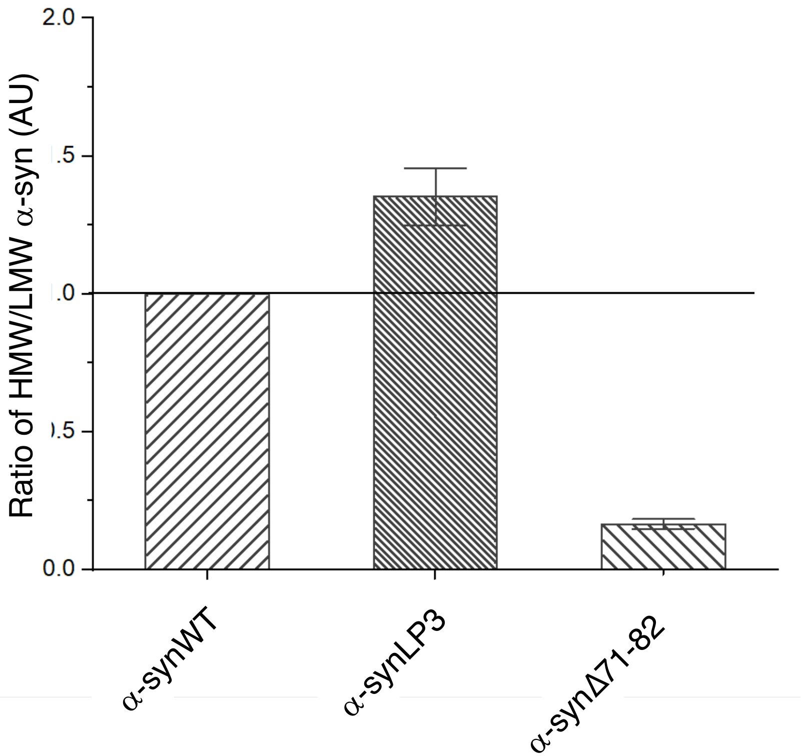

Figure S11: Deletion of the NAC region decreases the ratio of HMW/LMW α-syn species in native non-denaturing gel electrophoresis. Quantification of the ratio high molecular weight insoluble and low molecular weight soluble α-syn species from native non-denaturing gel electrophoresis of α-syn^{WT} and α-syn$^{\Delta 71\text{-}82}$ normalized to α-syn^{WT}. Note that while α-syn^{LP3} shows a increased ratio of HMW/LMW α-syn compared to α-syn^{WT}, α-syn$^{\Delta 71\text{-}82}$ shows a decreased ratio of HMW/LMW α-syn compared to α-syn^{WT}. n=2 independent membranes.

Figure S12:

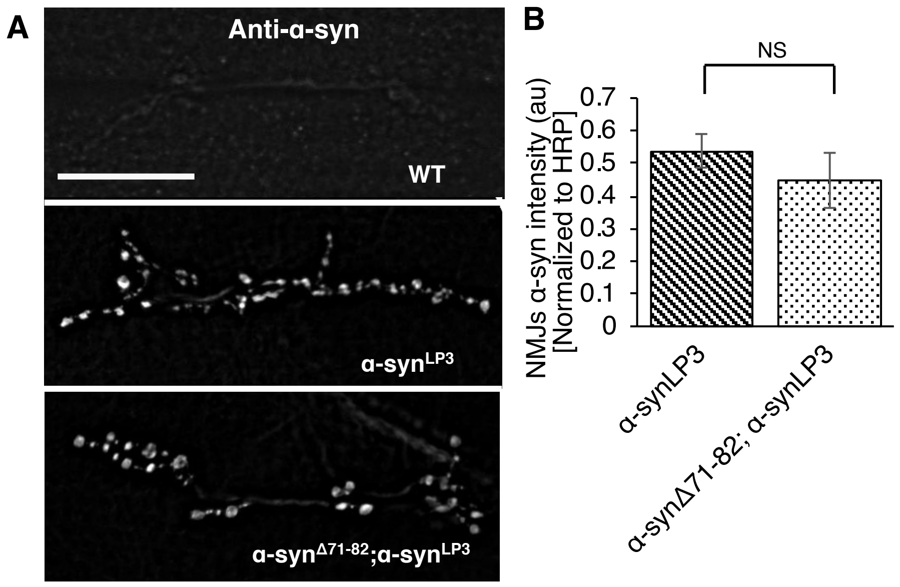

Figure S12: Expression of α-syn lacking the NAC region does not affect α-syn localization to larval NMJs. (A) α-syn and HRP staining in WT, α-syn^{LP3} alone and in larvae co-expressing α-syn^{LP3};α-syn$^{\Delta 71-82}$ larval NMJs at segment A2 of muscle 6/7 (Bar = 25µm). (**B**) Quantitative analysis indicates no significant change in α-syn intensity (normalized to HRP) at α-syn^{LP3};α-syn$^{\Delta 71-82}$ larval NMJs compared to α-syn^{LP2} larval NMJs ($p<0.05$, n= 10).

Figure S13:

A

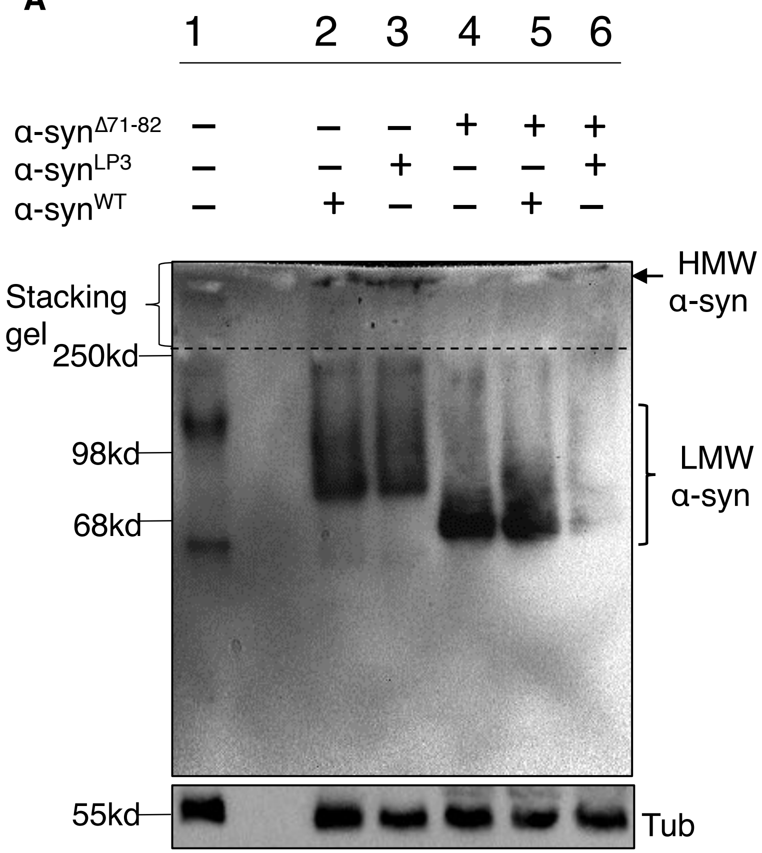

B

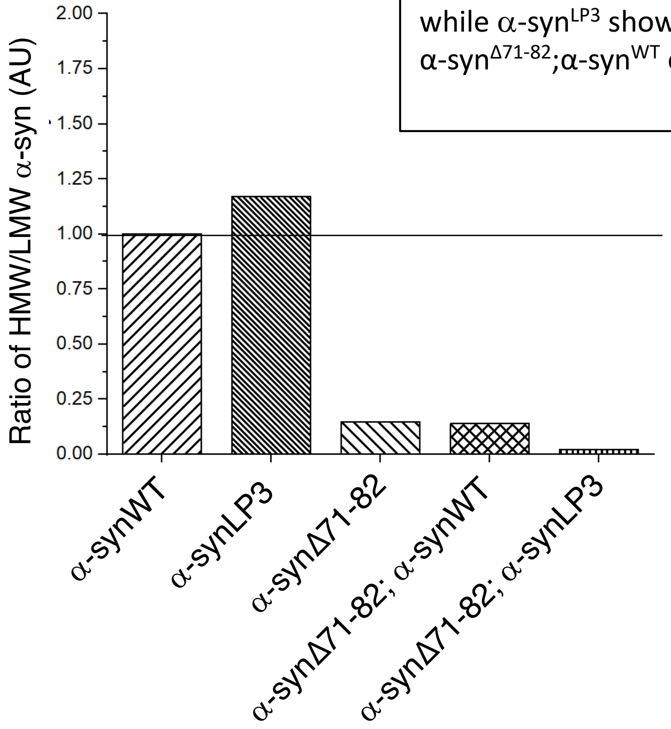

Figure S13: Expression of α-syn lacking the NAC region decreases the higher molecular weight species of α-syn. (A) Native gel electrophoresis and Western blot analysis of α-syn in α-syn$^{Δ71\text{-}82}$;α-syn^{WT} expressing flies (Lane 5) or α-syn$^{Δ71\text{-}82}$;α-syn^{LP3} (Lane 6) expressing flies show decreased or no HMW α-syn species (arrow) compared to α-syn^{WT} (Lane 2) or α-syn^{LP3} flies (Lane 3) respectively. Lane 1 is the WT control and Lane 2 is the α-syn$^{Δ71\text{-}82}$ expressing flies. Tubulin is used as a loading control. **(B)** Quantification of the ratio high molecular weight insoluble and low molecular weight soluble α-syn species from native non-denaturing gel electrophoresis of α-syn^{WT}, α-syn^{LP3}, α-syn$^{Δ71\text{-}82}$ alone and co-expressing α-syn$^{Δ71\text{-}82}$;α-syn^{WT} and α-syn$^{Δ71\text{-}82}$;α-syn^{LP3} normalized to α-syn^{WT}. Note that while α-syn^{LP3} shows an increased ratio of HMW/LMW, lines co-expressing α-syn$^{Δ71\text{-}82}$;α-syn^{WT} or α-syn$^{Δ71\text{-}82}$;α-syn^{LP3} show decreased ratios.

Figure S14:

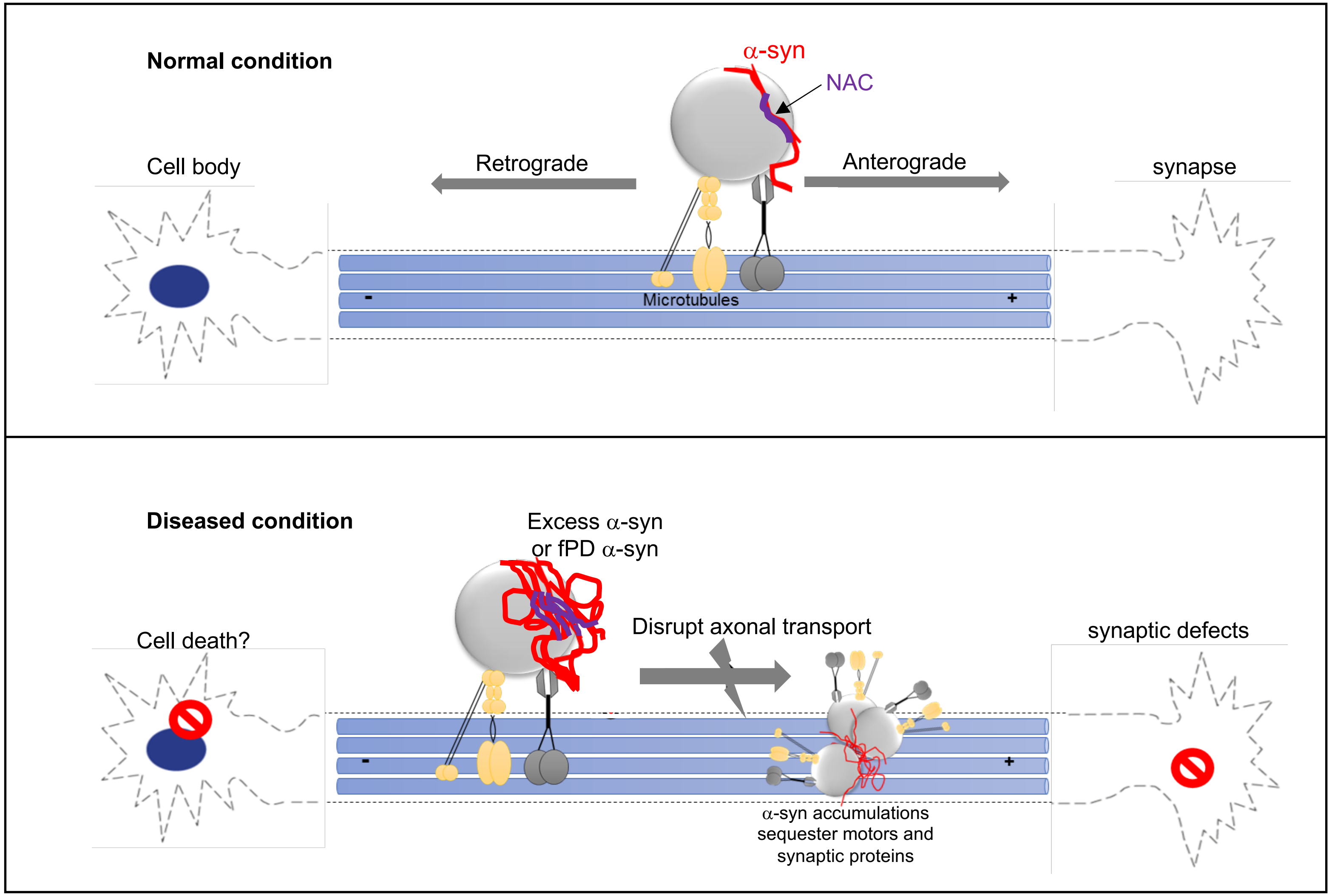

Figure S14: Schematic model of how too much or fPD mutant α-syn perturbs axonal transport via aggregate formation, which in turn instigate synaptic dysfunction and neuronal death. (**A**) Under normal conditions α-syn is transported bi-directionally with axons via associations with molecular motors bound to synaptic vesicles. **(B)** Under diseased conditions, too much or fPD mutant α-syn which is prone to form α-syn aggregates interfere with the axonal transport machinery disrupting the transport of essential synaptic cargo within axons. Decrease in the motility of synaptic cargo to nerve terminals cause synaptic dysfunction and behavioral defects which over time may instigate neuronal cell death at cell bodies.
